# Culture-space control is effective in promoting haploid cell formation and spermiogenesis in vitro in neonatal mice

**DOI:** 10.1038/s41598-023-39323-y

**Published:** 2023-07-31

**Authors:** Kiyoshi Hashimoto, Hisakazu Odaka, Yu Ishikawa-Yamauchi, Shino Nagata, Hiroko Nakamura, Hiroshi Kimura, Takuya Sato, Kazuhide Makiyama, Takehiko Ogawa

**Affiliations:** 1 Department of Urology, Graduate School of Medicine, Yokohama City University, Yokohama, Japan; 2 Department of Regenerative Medicine, Yokohama City University, Yokohama, Japan; 3 Graduate School of Medical Life Science, Yokohama City University, Yokohama, Japan; 4 Micro/Nano Technology Center, Tokai University, Hiratsuka, Japan

**Keywords:** Biological techniques, Developmental biology

## Abstract

The classical organ culture method, in which tissue is placed at the gas‒liquid interphase, is effective at inducing mouse spermatogenesis. However, due to reginal variations in the supply of oxygen and nutrients within a tissue, the progress of spermatogenesis was observed only in limited areas of a tissue. In addition, haploid cell formation and its differentiation to spermatozoon, i.e. spermiogenesis, were infrequent and inefficient. Here, we show that the polydimethylsiloxane (PDMS)-chip ceiling (PC) method, which ensures a uniform supply of nutrients and oxygen throughout the tissue by pressing it into a thin, flat shape, can provide control over the culture space. We used this method to culture testis tissue from neonatal mice, aged 1 to 4 days, and found that modulating the culture space during the experiment by replacing one chip with another that had a higher ceiling effectively increased tissue growth. This adjustment also induced more efficient spermatogenesis, with the process of spermiogenesis being particularly promoted. Meiotic cells were observed from culture day 14 onward, and haploid cells were confirmed at the end of each experiment. This technique was also shown to be a sensitive assay for testicular toxicity. Culture-space control will be a critical regulation parameter for sophisticated tissue culture experiments.

## Introduction

In 2011, our research group succeeded in mouse in vitro spermatogenesis using the air‒liquid interphase method, a classical organ culture technique in which testis tissue is placed on an agarose gel half-soaked in a culture medium^1^. We named this the original agarose gel (AG) method. The AG method is simple and has been reproduced in multiple laboratories, confirming its reproducibility^2–6^. However, the AG method has a disadvantage that is common to tissue culture experiments in general: the microenvironment inside the tissue is greatly heterogeneous due to an inevitable collapse of the microcirculatory system at explantation. This naturally degrades the efficiency of the in vitro spermatogenesis. Accordingly, we developed a chip-type device, made of polydimethylsiloxane (PDMS), that made it possible to equalize the supply of oxygen and nutrients to each seminiferous tubule by flattening the cultured tissue (PDMS-ceiling method: PC method). PDMS, with its high affinity for biological samples and high gas permeability, is widely used in the fabrication of microfluidic devices for cell culture and other applications. Using the PC chip, we have shown that it is possible to avoid the degeneration and necrosis of the central part of the tissue, which can occur in the AG method, thereby promoting the growth of testicular tissue and spermatogenesis^7,8^. In addition, since the height of the culture space is defined in the PC method, it is possible to measure the tissue volume during an experiment over time by measuring the horizontal projection area of the cultured tissue^7^.

In this study, we used double transgenic (Tg) mice harboring *Acrosin (Acr)*-GFP and H3.3-mCherry (mCherry) as transgenes to monitor the progress of spermatogenesis and to accurately evaluate spermiogenesis, i.e. the maturation of spermatids into sperm. In addition, taking advantage of the PC method, we monitored changes in tissue volume over time and investigated their relation to the progress of spermatogenesis. Then, we found that PC chip replacement during cultivation, which led to an expansion of culture space vertically, promoted spermatogenesis, especially spermiogenesis. Lastly, a synthetic estrogen, ethinyl estradiol (EE), was used as an inhibitor of spermatogenesis^9,10^ to show that this culture method is useful as a testicular toxicity test.

## Results

### Re-examination of classical organ culture method using agarose gel stand (AG method)

**Figure 1:**
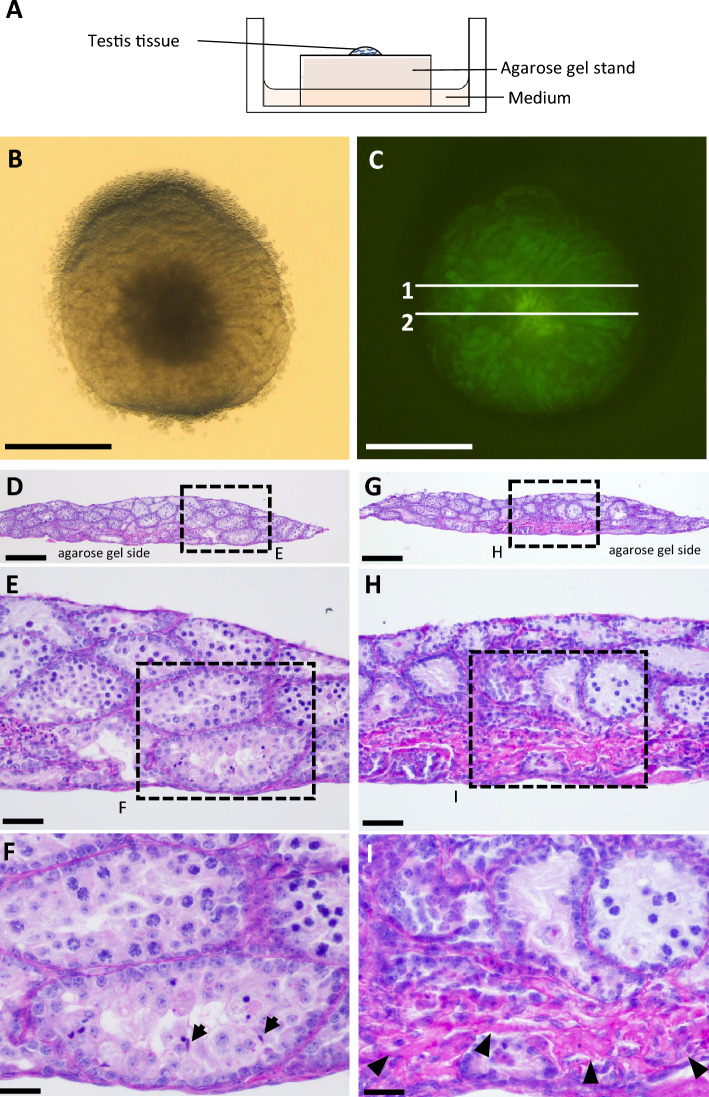
Re-examination of organ culture method using agarose gel stand (AG method). (**A**) Schematic drawing of AG method. (**B**,**C**) Stereomicroscopic picture of testis tissue from a 4-day-old mouse, cultured for 32 days. Bright-field (**B**) and GFP emission under excitation light (**C**). (**D**) PAS-stained histological view by vertical section, corresponding to line 1 in (**C**). (**E**) Enlarged picture of the dashed-line rectangle in (**D**). (**F**) Enlarged picture of the dashed-line rectangle in (**E**). Arrows indicate heads of elongated spermatids. (**G**) Vertical section of cultured tissue stained with PAS, corresponding to line 2 in (**C**). (**H**) Enlarged picture of the dashed-line rectangle in (**G**). (**I**) Enlarged picture of the dashed-line rectangle in (**H**). Arrow heads indicate necrotic areas corresponding to central necrosis of the tissue. Scale bar: 1 mm (**B**,**C**), 200 μm (**D**,**G**), 50 µm (**E**,**H**), 25 µm (**F**,**I**).

The AG method has been widely used in in vitro spermatogenesis studies because of its high reproducibility, simplicity, and efficiency in spermatogenesis induction (Fig. 1A). To monitor the progression of spermatogenesis in cultured tissues, we have been using testicular tissues from *Acr*-GFP Tg mice and measuring GFP expression under a stereomicroscope. In the present experiment using 4-day-old mouse testis tissue, extensive areas of seminiferous tubules expressed *Acr*-GFP when the sample was cultured by the AG method and observed from above with a stereomicroscope (Fig. 1B,C). However, when vertical sections of the cultured tissue were made, remarkably uneven distribution was observed in both germ cell numbers and the progression of spermatogenesis within each of the multilayered seminiferous tubules. Specifically, the seminiferous tubules near the agarose gel in the para-peripheral region of the cultured tissue contained spermatids, whereas in the seminiferous tubules facing or close to the gas phase, germ cell differentiation seemed stagnated at the early meiotic phase (Fig. 1D–F). In the central part of the cultured tissue, degeneration and necrosis of the seminiferous tubules were observed (Fig. 1G–I). Thin seminiferous tubules, devoid of germ cells and consisting only of Sertoli cells, were also observed. These observations highlighted the shortcomings and limitations of the AG method. We interpreted these results to mean that, in the AG method, oxygen and nutrients are not uniformly supplied to each part of each seminiferous tubule and that the microenvironment greatly differs from compartment to compartment within the tissue mass.

### Effect of culture-space height on testis tissue growth and spermatogenesis

**Figure 2:**
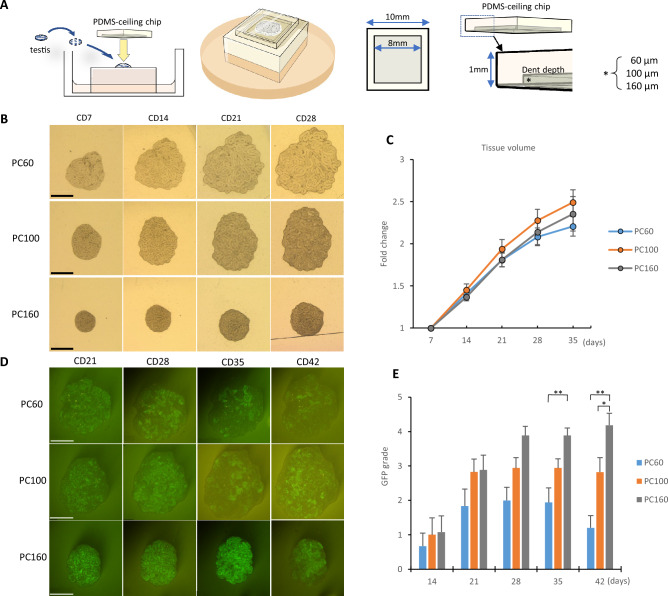
Effect of culture-space height on testis tissue growth and spermatogenesis. (**A**) Schematic presentation of the PC method. (**B**) Stereomicroscopic view of cultured tissues. (**C**) Relative tissue volume compared to the volume on culture day 7. There were 19–20 sample tissues for each group. No significant difference was observed among the three groups. (**D**) Stereomicroscopic view of cultured tissues under excitation light. GFP emission is visible in the seminiferous tubules. (**E**) Summary graph representing GFP grade in each PC group. There were 17–18 sample tissues in each PC group. Error bars indicate SEM. Scale bar: 1 mm (**B**,**D**). *p < 0.05, **p < 0.01.

To reduce the heterogeneity of the regional microenvironment in each cultured tissue observed in the AG method, we developed the PC method, in which the tissue is covered with a chip-type microdevice made of PDMS (Fig. 2A)^7,8^. The PC chip presses the testis tissue into a thin and flat shape, which can equalize the supply of nutrients and oxygen to the entire tissue and reduce or abolish the central necrosis that can occur in the AG method. In addition, tissue volume can be calculated sequentially in the PC method by measuring the horizontal projection area of the tissue.

In the PC method, the dent depth of the PC chip is an important variable in chip design because it defines the thickness of the tissue piece. In previous reports, tissue growth was higher under a PC chip whose dent depth was 60 µm or 100 µm in a short-term (1 week) culture^7^, whereas the efficiency of spermatogenesis was superior under a PC chip having 150 to 200 µm of dent depth in long-term culture^8^. Therefore, in the present study we used three sets of PC chips, which had dent depths of 60 µm, 100 µm, and 160 µm; these are hereafter called PC60, PC100, and PC160. To keep records of volume changes in the cultured tissues, stereomicroscopic photos were taken every week (Fig. 2B). The horizontal projection area of the tissue in the photograph was measured using image analysis software, and the tissue volume was calculated by multiplying the dent depth of the PC chip. To compare the degree of volume change between cultured tissues, the volume change rate of each tissue was calculated, using the volume on culture day 7 as the base point, by dividing each volume value by the base point value. The tissue volume increased during cultivation in every PC chip group, with no significant differences among the three groups (Fig. 2C). The efficiency of spermatogenesis was evaluated by the *Acr*-GFP expression area ratio. *Acr*-GFP fluorescence was observed from around 14 days of culture in all PC groups; its expression area ratio increased until 21 to 28 days of culture (Fig. 2D). To semi-quantify GFP expression, we used the GFP grading scale (Supplementary Fig. 1A) and summarized the data in a graph (Fig. 2E, Supplementary Fig. 3). The GFP grades were lower in the PC60 and PC100 groups than in the PC160 group, and in PC60 the grade tended to decline after day 35. On the other hand, the PC160 group maintained a high GFP grade throughout the culture period.

### Culture-space control by changing the dent depth

**Figure 3:**
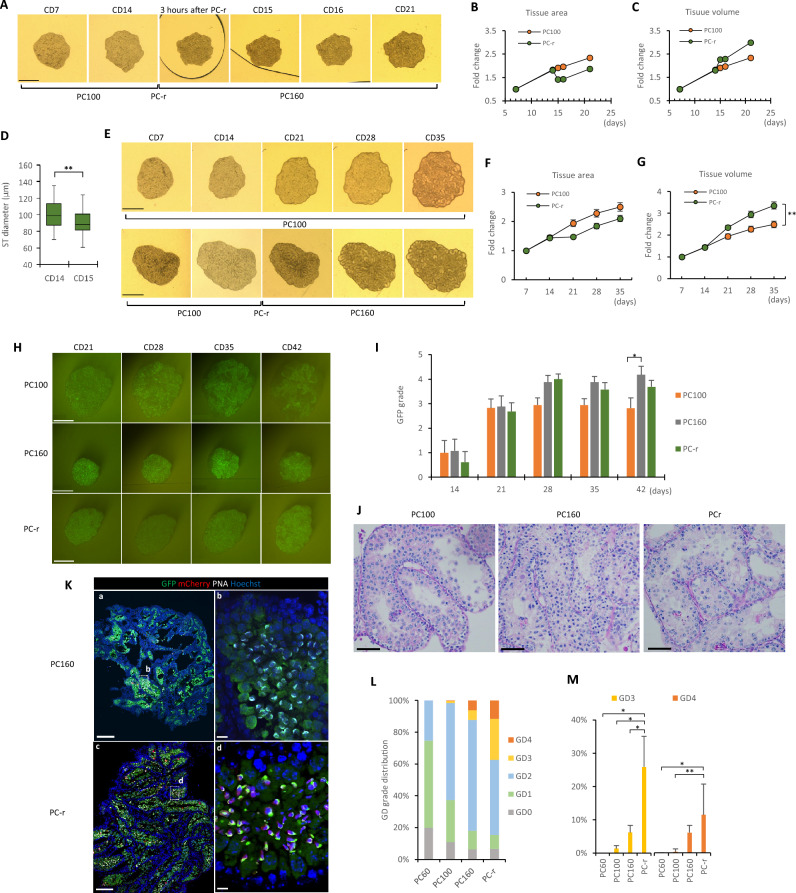
Culture-space control by replacing the PC chip. (**A**) Sequential stereomicroscopic view of a cultured tissue from culture day 7 to 21, with PC-chip replacement on day 14. (**B**) Horizontal projection area change rate of the tissue before and after PC-chip replacement. Data of three experiments of 9 sample tissues in each group were used. (**C**) Volume change rate of the tissue before and after PC-chip replacement. (**D**) Seminiferous tubule diameters measured with stereomicroscopic images of the tissue on culture day 14 (CD14; before PC-chip replacement) and day 15 (CD15). Data obtained in 3 experiments, 81 seminiferous tubules in 9 tissues, were used. (**E**) Sequential stereomicroscopic view of the cultured tissue by PC100 and PC-r. (**F**) Horizontal projection area change rate of the tissues in the PC100 (n = 20) and PC-r (n = 26) groups. No significant difference between groups. (**G**) Volume change rate of the tissues in PC100 (n = 20) and PC-r (n = 26) groups. (**H**) Stereomicroscopic view of cultured tissues under excitation light. GFP emission is visible in the seminiferous tubules. (**I**) Summary graph representing GFP grade in PC100, PC160, and PC-r. There were 17–19 sample tissues in each group. No significant difference was found among the groups. (**J**) Histological picture of tissues cultured with PC100, PC160, and PC-r for 42 days, stained with PAS. One day old mouse testis tissues were cultured for 42 days and examined. (**K**) Immunohistochemical staining of tissues in the PC160 and PC-r groups. GFP: green, mCherry: red, PNA: white, Hoechst: blue. Dashed-line frames are enlarged in the panel with the same letters, (**b**) and (**d**). Elongated spermatids are seen in the PC-r sample. (**L**) Summary graph of GD grade distribution in each group: PC60, PC100, PC160, and PC-r. Samples were analyzed on culture days 35 to 42 (corresponding to mouse ages of 36–43 dpp). (**M**) Rates of GD grades 3 and 4 were compared among the groups. There were 6, 8, 9, and 9 sample tissues in the PC60, PC100, PC160, and PC-r groups, respectively. The numbers of seminiferous tubules examined were 190, 377, 626, and 516, respectively. Error bars indicate SEM. Scale bar: 1 mm (**A**,**E**,**H**), 200 μm (**K**)-(**a**,**c**)), 50 μm (**J**), 10 μm (**K**)–(**b**,**d**). *p < 0.05, **p < 0.01.

From those inferior results for PC60 and PC100 compared to PC160, we hypothesized that the ceiling of the PC chip restricted the tissue’s vertical space, thus preventing the expansion of the seminiferous tubules, which in turn increased pressure in the tissue, and that the ceiling inhibited the proliferation and differentiation of the germ cells inside. We then came up with an idea to raise the ceiling to increase the culture space in order to accommodate the physiological growth of the seminiferous tubules. We conducted an experiment in which we used PC100 initially and changed to PC160 on culture day 14; we called this the PC-replacement (PC-r) group. We carefully observed the tissues after the replacement of the PC chip to observe the effect of the change in height. After the replacement, the horizontally projected area of the cultured tissue shrank to the minimum in as little as 3 h (Fig. 3A,B). This seemed to reflect the ceiling elevation causing both a vertical expansion and a horizontal contraction of the tissue, maintaining the volume almost constant (Fig. 3C). Consistent with these changes in the tissue as a whole mass, the seminiferous tubules had smaller diameters on day 15 than on day 14 (Fig. 3D). This suggested that on day 14 the seminiferous tubules were pressed and elliptical under the PC100 chip. The horizontal projection area of the tissues showed a slight increase on day 16 and increased gradually thereafter, as the nonreplaced PC100 group did during the following 3 weeks (Fig. 3E,F). Naturally, the tissue volume in the PC-r group increased more significantly than in the PC100 group (Fig. 3G). The volume expansion of samples was more prominent in PC-r than in other groups (Supplementary Fig. 2). Given that mouse Sertoli cells cease their proliferation by around day 14 after birth^11^, we supposed that the subsequent volume increase reflected the increased number of germ cells, including the appearance of meiotic spermatocytes, and the accumulation of intratubular fluid.

As for *Acr*-GFP expression, the PC-r group maintained GFP grades throughout the culture period that were as high as those in the PC160 group (Fig. 3H,I and Supplementary Fig. 3). Regular histological examination with PAS staining at the end of experiment on culture day 42 showed layers of spermatogenic cells, including meiotic cells, in the seminiferous tubules in all experimental groups, confirming that Acr-GFP expression were corresponding to the presence of meiotic cells (Fig. 3J). To evaluate the progression of spermatogenesis in detail, we used immunohistochemical staining, by which seminiferous tubules were classified into five grades according to the differentiation stage of the germ cells they contained (germ cell differentiation grade: GD grade). The GD grade was determined by immunohistochemical images with anti-GFP antibody, Peanut agglutinin (PNA), and anti-RFP (mCherry) antibody (Supplementary Fig. 1B). In the PC60 and PC100 groups, seminiferous tubules classified as GD grades 1 and 2 were observed, but it was rare to find tubules classifiable as GD grade 3 or 4, suggesting that the germ cell differentiation stagnated around meiotic division or in the early stage of spermiogenesis. In the PC160 group, seminiferous tubules containing round and elongated spermatids were observed, though not in large numbers, i.e. GD grades 3 and 4 were 6.2% and 6.1%, respectively. In the PC-r group, on the other hand, the haploid cell formation and maturation were markedly accelerated; GD grades 3 and 4 were 25.9% and 11.5%, respectively (Fig. 3K–M). These results suggested that changes in the testis tissue volume and seminiferous tubule diameter in our culture system were closely related to the progression of spermatogenesis, especially spermiogenesis.

### Optimal oxygen concentration with PC-r method

**Figure 4:**
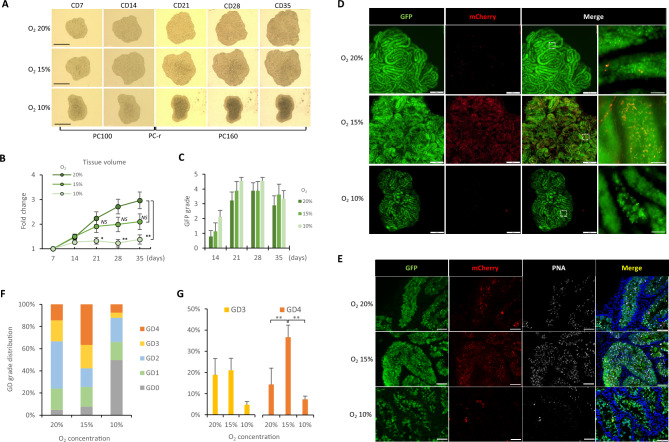
Optimal oxygen concentration with the PC-r method. (**A**) Sequential stereomicroscopic view of cultured tissues with the PC-r method under O_2_ concentrations of 20%, 15%, and 10%. (**B**) Relative volume change during the cultivation under each O_2_ concentration. Data of three experiments with 11–12 sample tissues in total in each group were used. (**C**) GFP grades of the cultured testis tissues under different O_2_ concentrations. There were 8–9 sample tissues in each group. No significant difference was found among the groups. (**D**) Microscopic view of fluorescence emissions, *Acr*-GFP and H3.3-mCherry, in tissues cultured under different O_2_ concentrations. (**E**) Immunohistochemical staining of tissues cultured under different O_2_ concentrations. GFP: green, mCherry: red, PNA: white, Hoechst: blue. (**F**) Summary bar graph of GD grade distribution in each group. There were 11, 12, and 9 sample tissues in the 20%O_2_, 15%O_2_, and 10%O_2_ concentrations, respectively. The exact numbers of seminiferous tubules examined were 773, 860, and 501 in the 20% O_2_, 15% O_2_, and 10% O_2_ concentrations, respectively. (**G**) Rates of GD grades 3 and 4 were compared among the groups. Error bars indicate SEM. Scale bar: 1 mm (**A**), 500 μm (**D**), 50 μm (**D**-right image in Merge), 50 μm (**E**). *p < 0.05, **p < 0.01.

Previously we reported the importance of oxygen concentration in spermatogenesis in vitro^12,13^. Here, we conducted an experiment to investigate the optimal oxygen concentration for PC-r. We set the oxygen concentrations in the incubator at 20%, 15%, and 10%, and assessed the tissue volume change and spermatogenic efficiency at each concentration. The tissue volume increased most significantly under 20% O_2_, followed by 15% O_2_ (Fig. 4A,B). Under 10% O_2_, tissues showed almost no increase in volume, and some showed a darkened central area, suggesting a shortage of oxygen for supporting cell proliferation (Fig. 4A). When spermatogenic efficiency was compared by GFP grade, no significant differences were observed among the three groups (Fig. 4C). As for mCherry, on the contrary, it was expressed widely in tissues cultured under 15% O_2_, while only sporadic mCherry-positive cells were observed in the other groups (Fig. 4D). Immunohistochemical staining showed that 36.6% of the seminiferous tubules (315/860) contained elongated spermatids (GD grade 4) under 15% O_2_ and 17.1% (70/409) under 20% O_2_, indicating that 15% O_2_ resulted in higher efficiency in haploid formation (Fig. 4E,F). Interestingly, at the 15% and 20% O_2_ concentrations, the percentages of GD grades 2 to 4 combined were almost the same, and the advantage of 15% O_2_ was restricted to the increase of GD grades 3 and 4. In particular, the percentage of GD grade 4 was significantly higher in 15% O_2_ group (Fig. 4G), suggesting that this oxygen concentration was particularly beneficial for promoting spermiogenesis. Under 10% O_2_, seminiferous tubules of GD grade 3 or 4 were occasionally observed at peripheral regions of the tissue, but nearly half of the seminiferous tubules had few or no germ cells.

### Efficacy of PC-r as a reproductive toxicity test

**Figure 5:**
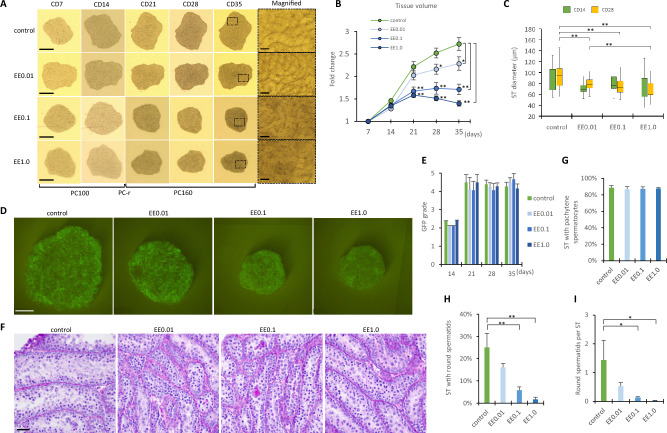
Toxicity of ethinyl estradiol (EE) to mouse testis evaluated with the PC-r method. (**A**) Sequential stereomicroscopic view of cultured tissues with different concentrations of EE. Pictures in the rightmost row show enlarged views of dashed-line frames in the left panels. (**B**) Relative volume of cultured tissue in each group from days 7 to 35 (n = 5 in EE0.01 and 12 in each of the other groups). (**C**) Diameter of the seminiferous tubules at each EE concentration on culture days 14 and 28. There were 8–9 tissue samples and 70–81 seminiferous tubules in each group. (**D**) Stereomicroscopic view of cultured tissues under GFP-excitation light on culture day 35, with different concentrations of EE exposed. (**E**) GFP grades of cultured tissues under different EE concentrations from days 14 to 35. There were 8 to 15 tissue samples in each group. No significant difference was found among the groups. (**F**) Representative histological figures of testis tissues cultured for 35 days under different EE concentrations with PAS staining. (**G**) Percentage of seminiferous tubules containing pachytene spermatocytes in histological sections. No significant difference was found among the groups. (**H**) Percentage of seminiferous tubules containing round spermatids. (**I**) Number of haploid cells per seminiferous tubule of cultured tissue in a histological specimen. To obtain data in graphs (**G**, **H** and **I**), two experiments were performed, with 5 testis tissues cultured in each experimental group. The numbers of examined seminiferous tubules were 858, 522, 550, and 482 in the control, EE0.01, EE0.1, and EE1.0 groups, respectively. Error bars indicate SEM. Scale bar: 1 mm (**A**,**D**). 50 μm ((**A**): image of right side, (**F**)). *p < 0.05, **p < 0.01.

Culturing testis tissues using the PC-r method under 15% O_2_ was shown to induce spermatogenesis, including spermiogenesis, most efficiently. We reasoned therefore that this culture system would serve well for toxicity testing. Using the PC-r method, we then conducted a testicular toxicity test in which the test chemical was EE, a synthetic estrogen with confirmed testicular toxicity^9,10,14–19^. EE was added to the culture medium at 0.01, 0.1, and 1.0 nM, and the same amount of a solvent, DMSO, were added in the control group. During the first 2 weeks, no apparent differences were noticed among the experimental groups, but the tissue volume expansion rate revealed significant differences later on (Fig. 5A). The volume expansion was reduced in an EE-dose-dependent manner, and the difference was significant compared to the control as early as culture day 21 (Fig. 5B). The mean diameters of the seminiferous tubules were all smaller in the EE-administered groups than in the control group, and the difference was significant on culture day 28 (Fig. 5C). Regarding spermatogenic progression, there was no significant difference in the GFP grade among the groups regardless of the EE concentrations (Fig. 5D,E). Correspondingly, histological examination with PAS staining showed no significant difference among groups in the percentage of seminiferous tubules containing pachytene spermatocytes (Fig. 5F,G). This suggested that the toxicity of EE has little effect on germ cells up to the meiotic prophase stage. On the contrary, however, the percentage of seminiferous tubules containing round spermatids decreased significantly in the EE 0.1 nM and 1.0 nM groups, indicating that EE inhibited the differentiation to haploid cells (Fig. 5H). Also, there were significantly fewer round spermatids per seminiferous tubule in the EE 0.1 nM and 1.0 nM groups (Fig. 5I). Taken together, these results suggested that the volume change in the cultured testicular tissue in the PC-r method reflected a disturbance of spermatogenesis, i.e. an inhibition of haploid formation, in the seminiferous tubules in a reliable and sensitive manner. This finding indicates the potential ability to predict the efficiency of spermatogenesis only by monitoring volume changes in cultured tissue. Importantly, this can be done without markers of spermatogenesis found in Tg mice, such as Acr-GFP or mCherry; i.e. it can even be done in wild-type mice.

## Discussion

Since the success of in vitro spermatogenesis in mice in 2011, many researchers, including ourselves, have improved the system by modifying culture media and developing new culture devices^20–30^. We introduced a microfluidic system (MFD) into the testis tissue culture experiment, created a culture space by silicone resin PDMS, which is highly oxygen permeable, and made a flow of culture medium beside the tissue, in order to improve the efficiency of spermatogenesis and maintain it for a long term, as long as half a year^20,24^. On the other hand, in the course of this experience, we realized that the processing of multiple specimens, which was possible with the AG method, was difficult with MFD, making the experiment inefficient in terms of labor and cost. At the same time, we recognized that the advantage of using PDMS is that it makes the tissue thinner and flatter, facilitating the molecular exchange of oxygen, nutrients, and waste products in every corner of the entire tissue. On this basis, we developed the PC method, whose advantages we demonstrated in our previous studies^7,8^. In using the PC method, an important parameter we have to choose is the height of the culture space, which is defined by the depth of the dent in the PC chip. Theoretically, if the dent is shallow, i.e. if the height of the culture space shrinks and the tissue becomes thinner, molecular exchange (the supply of nutrients and oxygen and the removal of waste) will be efficient. However, a dent too shallow leads to too much compression of the seminiferous tubules, thus impairing their growth and function. In this regard, the present study showed that PC dent depths of 60 µm and 100 µm were in the region where the benefit and the damage almost balance each other. The culture spaces defined by these depths were appropriate for neonatal tissue for a while, but too narrow for their growth and spermatogenesis. Here, it is noteworthy that using a PC chip to limit the culture space enabled us to control the space volume and shape. Therefore, we devised a strategy to expand the culture space by changing the PC chip for one that had a dent deep enough to accommodate the growth of the tissue and the thickening of the seminiferous tubules, i.e. the progress of spermatogenesis. In this study, we replaced the PC chip during experiments to ones that would increase the height of the culture space from 100 to 160 µm. The effect was greater than we expected and resulted in the remarkable promotion of spermatid maturation. It is interesting to see new experiments such as space expansion in a more precise manner, or to see periodic changes in space volume from expansion to narrowing and narrowing to expansion, which could control spermatogenesis by means of physical commands. Such data should encourage the development of new culture devices that allow flexible and precise control of the culture space.

Experiments investigating the optimal oxygen concentration using PC-r showed the superiority of 15% O_2_ among the three conditions. Under 10% O_2_, tissue growth was significantly reduced, suggesting a shortage of oxygen. As the tissue growth was reduced as early as the second week, it was speculated that not only germ cells but also somatic cells suffered from an oxygen shortage. On the other hand, as for the 15% and 20% O_2_, seminiferous tubules classified as GD grades 2 to 4 in total showed almost the same rate in both groups. The superiority of 15% O_2_ was exclusively in increasing the rate of GD3 and 4, i.e. in promoting spermiogenesis. In the body, seminiferous tubules are surrounded by capillaries, forming an oxygen concentration gradient in the tubule from high in the periphery to low in the center. Therefore, spermiogenesis, which takes place in a more central region in the seminiferous tubule than meiosis or spermatogonial proliferation, would favor a lower O_2_ condition compared to the other two processes. The present results make sense in light of such subtle local O_2_ gradients in the animal body.

Recently, several studies reported the usefulness of an organ culture system, using prepubertal mouse testis tissue, as a method of evaluating the toxic nature of anticancer drugs cyclophosphamide, cisplatin, and doxorubicin^31,32^. At concentrations clinically used for pediatric patients, all these drugs induced significant germ cell loss, including that of spermatogonial stem cells, resulting in a Sertoli cell‒only state in most of the seminiferous tubules of cultured tissues. On the contrary to these anticancer drugs, which cause the total depletion of germ cells, EE showed mild toxicity that was limited to the later stage of spermatogenesis. In fact, EE is a synthetic estrogen, having an estrogenic effect almost equivalent to that of 17β-estradiol, and is frequently used as a contraceptive pill, meaning it has very minor, if any, adverse effects on women of reproductive age. However, due to its long half-life, there are concerns about its uptake into plants and aquatic animals, which in turn might contaminate food consumed by humans^9^. In experiments in adult rats, EE administration induced reductions in reproductive organ weight and fertility dose-dependently^10,14^. It has been also reported that exposure to EE in the neonatal^15^ and fetal periods^16,17^ could cause subsequent male infertility or subfertility, raising warnings of its toxicity to humans^18,19^. Our present study successfully demonstrated the testicular toxicity of EE even at a lower concentration of 0.01 nM, which indicates that the PC-r method was more sensitive than the AG method as a testicular toxicity test^33^. This owes to the superiority of the PC-r method in promoting the later stages of spermatogenesis, i.e. spermiogenesis, than the AG method or the conventional PC method. Moreover, it is noteworthy that the measurement of cultured tissue volume and seminiferous tubule diameter over time, which is possible with the PC method, can be a sensitive indicator for monitoring testicular toxicity. In animal experiments, testis weight and seminiferous tubule diameter have been used as important indicators for the evaluation of testicular toxicity^34,35^. However, the measurement required the sacrifice of experimental animals every time. Our study showed for the first time that measuring the volume of cultured testis tissue can be an alternative to measuring testis weight. It also endows additional benefits, i.e. it significantly reduces the number of animals used and makes it possible to measure the volume of a tissue sequentially over time. In addition, this evaluation system is highly versatile because it can be performed using wild-type mice with minimal experimental equipment.

In this study, we exclusively used immature mouse testis tissues. It is noteworthy to mention that a significant challenge in testis tissue culture experiments lies in the difficulty of working with adult mouse testis tissues as source samples, compared to immature ones. Maintaining ongoing spermatogenesis becomes impossible when a testis tissue piece is explanted from an adult mouse. We have only observed limited regeneration of spermatogenesis in such samples after the spermatogenic germ cells in the seminiferous tubules had disappeared^36^. Consequently, adult animals are not suitable for experiments that require quantitative in vitro spermatogenesis, such as the toxicity tests demonstrated in this study. Therefore, further improvements in the methods for culturing adult testis tissue are necessary.

Through the use of the PC-r method, this study demonstrated the importance of space control for cultured tissue for both tissue growth and the promotion of spermatogenesis. Therefore, space control is expected to become an important parameter in future research using organ cultures, and more sophisticated means of controlling the shape and volume of the culture space will be required.

## Methods

### Animals

*Acrosin (Acr)*-GFP transgenic mice^37^ (genetic background: C57BL/6) were provided by RIKEN BRC through the National Bio-Resource Project of MEXT, Japan. Histone H3.3-mCherry transgenic mice were provided by Makino & Okada (genetic background: C57BL/6)^38^. The *Acr*-GFP Tg line and the H3.3-mCherry Tg line were each crossed with wild-type ICR or C57BL/6 mice occasionally and maintained independently as *Acr*-GFP mice and H3.3-mCherry mice, whose genetic backgrounds were thus a mixture of ICR and C57BL/6. *Acr*-GFP/H3.3-mCherry double (*Acr*/H3.3) transgenic mice were generated by crossing a female *Acr*-GFP homogeneous mouse with a male H3.3-mCherry homogeneous mouse. Mice aged 1.5 to 4.5 days postpartum (dpp) were used as the source of testis tissue. Specifically, data in Figs. 1, 2, 3, 4 and 5 were obtained using 4, 1, 4, and 3–4 dpp mice, respectively. GFP expression in the spermatogenic cells of *Acr*-GFP transgenic mice starts from pachytene spermatocytes at stage 4 onward^39^. In histone H3.3-mCherry transgenic mice, mCherry is expressed in spermatids from step 11 onward^38^. Mice were housed in specific pathogen-free, air-conditioned rooms at 24 ± 1 °C and 55 ± 5% humidity, with a 13-h light/11-h dark lighting cycle. They were fed ad libitum with commercially available hard pellets (MF; Oriental Yeast). Drinking water was acidified to pH 2.8–3.0 by HCl. All animal experiments conformed to the Guide for the Care and Use of Laboratory Animals and were approved by the Institutional Committee of Laboratory Animal Experimentation (Animal Research Center of Yokohama City University, Yokohama, Japan) (protocol nos. F-A-20-038). The study is reported in accordance with ARRIVE guidelines.

### Culture media and reagents

To make culture medium, α‐modified Eagle Minimum Essential Medium (αMEM; Gibco: 12000‐022) was dissolved in water purified with Milli-Q (Millipore) at a twofold concentration, and AlbuMAX (Gibco: 11020‐021) was added at 40 mg/mL as a final concentration. Then, 7% NaHCO_3_ solution was added (0.026 ml/L) to achieve a final concentration of 1.82 g/L. Antibiotic‒antimycotic (15240062, Thermo Fisher Scientific) was added at a 1/100 volume to achieve a final concentration of 100 IU/ml for penicillin, 100 μg/mL for streptomycin, and 250 ng/ml for amphotericin. Lastly, the medium solution was brought to the final volume with MilliQ water. The medium was sterilized with a Millipore filter having a pore size of 0.22 µm and stored at 4 °C. In the testicular toxicity experiment, EE (10006486, Cayman Chemical Company) dissolved in DMSO (D5879, Sigma-Aldrich) was added to the culture medium at the concentrations indicated in the text. In every group, including the control, the amount of DMSO added was 1/10,000 (v/v) of the medium.

### PDMS ceiling chip

The PDMS ceiling (PC) chip was produced by mixing PDMS prepolymer and a curing reagent (Silpot 184; Dow Corning) at an 11:1 weight ratio. The mixture was poured over a mold master, placed in a vacuum chamber for degassing, and moved to an oven to be heated at 72 °C for 1.5 h for curing. After cooling, the solidified PDMS was peeled off from the master. This PDMS disk was diced into individual chips using a cutter knife. For sterilization of the PC chip, they were exposed to UV light for over one hour before use. The mold master was produced as previously reported using conventional photolithography and soft lithography techniques^7,40,41^.

Briefly, a negative‐type photoresist material (SU‐82100 & 2075; MicroChem Co.) was applied to a 4‐inch wafer via spin coating to achieve uniform thicknesses of 60, 100, and 160 μm. After prebaking process, ultraviolet light was shone through a photomask to delineate a cultivation area, followed by postbaking. The baked mold master was then developed by incubation in propylene glycol monomethylether acetate (GODO, Tokyo, Japan) for 20–30 min, followed by rinsing in isopropanol (code 32435‐70; Kanto Chemical, Kanto, Japan). This SU‐8 mold master can be used repeatedly for the replica molding of PC chips. All mold masters were fabricated by the Micro/Nano Technology Center at Tokai University.

### Culture method

Testes were decapsulated and divided with forceps into three pieces. They were approximately 0.1mm^3^ in volume for the 1 dpp mice and 0.15 mm^3^ for the 4 dpp mice. The tissue fragments were then placed on blocks of agarose gel in the wells of a 12-well culture plate (665180, Greiner Bio-One). To make the agarose gel block, agarose powder (346-00072, Dojindo) was dissolved in water purified with Milli-Q at 1.5% (w/v) and autoclaved. During cooling, 33 mL of agarose solution was poured into 10 cm dishes to form a 5-mm-thick gel. The gel was cut into approximately 12 mm square pieces, which were used as stands for testis tissue placement. Gels were submerged in culture medium in the 12-well culture plates for more than 6 h, twice, with a medium change in between. After the second medium removal, 0.5 ml of new medium was added to each well to half-soak the gels, upon which testis tissues were placed. In cases of the AG method, each gel stand was loaded with 3 tissues. In the PC method, a single tissue was placed on a gel and a PC chip having a dent depth of 60, 100, or 160 μm was placed over the tissue. The medium was changed once a week and was poured up to around half the height of the agarose gel (approximately 0.5 mL/well). The culture incubator was supplied with 5% carbon dioxide in air and maintained at 34 °C. In the oxygen concentration experiment, three incubators were used to set the concentration to 10%, 15%, and 20%, respectively.

### Observations

Cultured tissues were observed at least once a week under a stereomicroscope equipped with an excitation light for GFP (LeicaM205 FA; Leica). For semi-quantitative evaluation of *Acr*-GFP expression, cultured testis tissues were classified into six grades according to the rate of GFP-positive area: 0%, 1–20%, 21–40%, 41–60%, 61–80%, and 81–100%^30^. This GFP grading scale faithfully corresponded to spermatogenic progression, as confirmed in previous studies^1,20^. The horizontal projection area of the tissue was measured using Fiji, an open-source platform for biological-image analysis^42^. The volume of tissue was calculated by multiplying the measured horizontal projection area by the depth of the PC chip dent. The relative tissue area and volume in each cultured tissue were compared to those on culture day 7. In measuring the diameter of the seminiferous tubule, each tissue was divided into nine grids and a representative tubule was selected from each site.

### Histological and immunohistochemical examinations

In histological examinations, specimens were fixed with Bouin’s fixative and embedded in paraffin. A section for each specimen was stained with hematoxylin and periodic acid Schiff (PAS). The germ cell stage was evaluated according to morphology and the staining pattern of the acrosome and nucleus. In immunofluorescence staining, tissues were fixed with 4% paraformaldehyde in PBS at 4 °C overnight, then soaked in solutions of 10, 15, and 20% (w/v) sucrose in PBS for 1 h each in succession for cryoprotection. They were cryo-embedded in OCT compound (Sakura Finetek Japan) and cut into 7-μm-thick sections. The cryosections were washed with 0.2% PBT (0.2% Triton X–100 in PBS) for 10 min four times and then treated with 5% BSA in 0.2% PBT for 30 min. Incubation with primary antibodies in 0.2% PBT containing 5% BSA was performed overnight at 4 °C, followed by rinsing four times with PBS for 10 min each, after which secondary antibodies diluted in 0.2% PBT containing 5% BSA were applied for 1 h at room temperature. The sections were washed with PBS, and the nuclei were counterstained with Hoechst33342 dye (346-07951 Dojindo). Finally, sections were mounted with Prolong Diamond Antifade Mountant (Thermo Fisher Scientific) before observation. The primary antibodies were anti-GFP (1:1000, 04404-26, Nacalai Tesque) and anti-RFP (1:1000, PM005, MBL). Lectin Peanut agglutinin (PNA) from *Arachis hypogaea* (peanut), Alexa Fluor 647 Conjugate (1:500, L32460, Invitrogen), was used to identify acrosomes. The secondary antibodies were Alexa Fluor 488-conjugated donkey anti-rat antibody (1:200, A21208, Invitrogen) and Alexa Fluor 555-conjugated donkey anti-rabbit antibody (1:200, A31572, Invitrogen). Immunostained samples were observed with an inverted microscope (IX73, Olympus) or a confocal microscope (FV1000–MPE, Olympus). According to the morphologies and immunostaining patterns of nuclei and acrosomes, the seminiferous tubules with them were classified into five grades as shown in Supplementary Fig. 1B (germ cell differentiation grade; GD grade).

### Statistical analysis

Results are presented as means ± standard error (SE). Statistical analysis was performed using one‐way ANOVA followed by Student’s *t* test or the Tukey‒Kramer HSD test. *P* < 0.01 and *P* < 0.05 were considered to indicate statistical significance.

## Supplementary Information

Supplementary Information 1.

Supplementary Information 2.

Supplementary Information 3.

Supplementary Information 4.

Supplementary Information 5.

Supplementary Information 6.

Supplementary Information 7.

Supplementary Information 8.

Supplementary Information 9.

Supplementary Information 10.

Supplementary Information 11.

Supplementary Information 12.

Supplementary Figures.

## Data Availability

All data generated or analyzed during this study are included in this published article and its supplementary information files. Correspondence and requests for raw data and materials should be addressed to T.O.
