# Supplementary material for: Culture-space control is effective in promoting haploid cell formation and spermiogenesis in vitro in neonatal mice
Source: Sci Rep. 2023 Jul 31;13:12354. doi: 10.1038/s41598-023-39323-y (PMC10390558; doi:10.1038/s41598-023-39323-y)
Supplement: Supplementary file 1 — Supplementary Information 1. [file 41598_2023_39323_MOESM1_ESM.pdf]

GFP grade for each PC-chip

	CD14	CD21	CD28	CD35	CD42
PC60	0	0	4	5	2
PC60	0	0	4	5	
PC60	0	0	3	4	0
PC60	0	1	1	1	1
PC60	0	5	3		
PC60	0	4	4	2	
PC60	1	4	4	1	
PC60	1	5	5	4	
PC60	0	1	1	1	1
PC60	0	1	1	0	1
PC60	5	5	1	1	1
PC60	5	5	1	4	4
PC60	0	1	1	1	
PC60	0	0	0	1	
PC60	0	1	1	2	
PC60	0	0	1	1	1
PC60	0	0	1	0	1
PC60	0	0	0	0	0
PC100		3	4	4	3
PC100		1	2	3	2
PC100		1	2	2	1
PC100		1	2	1	2
PC100	0	3	4	2	
PC100	3	3	4	4	
PC100	1	5	5	5	
PC100	0	1	4	4	3
PC100	0	1	2	2	2
PC100	5	5	1	3	5
PC100	4	5	2	4	5
PC100	0	3	2	3	
PC100	0	4	4	4	
PC100	0	3	1	2	
PC100	0	4	4	2	4
PC100	0	1	3	3	1
PC100	0	4	4	2	3
PC160		1	3	2	2
PC160		1	2	3	2

PC160		1	2	2	4
PC160		1	3	4	5
PC160	1	5	5	3	
PC160	2	5	5	5	
PC160	2	5	5	4	
PC160	0	1	4	4	5
PC160	0	1	4	4	5
PC160	4	5	5	5	5
PC160	5	5	5	5	5
PC160	0	1	4	4	
PC160	0	4	5	5	
PC160	0	3	4	4	
PC160	0	4	4	4	4
PC160	0	2	2	4	4
PC160	0	4	4	4	5
PC-r	0	3	4	2	
PC-r	0	4	5	4	
PC-r	0	5	5	4	
PC-r	0	2	4	5	4
PC-r	0	1	2	1	3
PC-r		1	2	2	3
PC-r		1	4	2	2
PC-r		1	4	3	3
PC-r		1	3	3	4
PC-r		2	5	5	5
PC-r		1	4	4	3
PC-r	3	5	5	5	5
PC-r	5	5	5	5	5
PC-r	0	4	4	4	
PC-r	0	4	5	5	
PC-r	0	3	3	4	
PC-r	0	4	4	3	4
PC-r	0	2	4	3	3
PC-r	0	2	4	4	4
