# Supplementary material for: Culture-space control is effective in promoting haploid cell formation and spermiogenesis in vitro in neonatal mice
Source: Sci Rep. 2023 Jul 31;13:12354. doi: 10.1038/s41598-023-39323-y (PMC10390558; doi:10.1038/s41598-023-39323-y)
Supplement: Supplementary file 2 — Supplementary Information 2. [file 41598_2023_39323_MOESM2_ESM.pdf]

Change rate of the tissue before and after PC-chip replacement.

	area(mm2)					area rate			
	CD7	CD14	CD15	CD16	CD21	CD7	CD14	CD15	CD16
PC100	2.02	3.16	3.3	3.42	4.28	1	1.56	1.63	1.69
PC100	0.89	1.23	1.3	1.34	1.56	1	1.38	1.46	1.51
PC100	1.3	1.88	1.97	2.04	2.35	1	1.45	1.52	1.57
PC100	1.62	4.28	4.52	4.66	5.55	1	2.64	2.79	2.88
PC100	2.27	5.96	6.01	6.15	7.72	1	2.63	2.65	2.71
PC100	1.74	4.39	4.55	4.77	5.79	1	2.52	2.61	2.74
PC100	4.8	7.05	7.45	7.58	8.63	1	1.47	1.55	1.58
PC100	4.89	6.73	7.08	7.15	7.8	1	1.38	1.45	1.46
PC100	5.46	8.24	8.63	8.69	10.1	1	1.51	1.58	1.59
PCr	2.11	3.31	2.7	2.9	4.01	1	1.57	1.28	1.37
PCr	2.23	3.53	2.89	3.04	4.44	1	1.58	1.3	1.36
PCr	1.54	2.29	1.79	1.84	2.09	1	1.49	1.16	1.19
PCr	1.43	3.39	2.58	2.58	3.25	1	2.37	1.8	1.8
PCr	1.2	2.97	2.21	2.23	2.91	1	2.48	1.84	1.86
PCr	1.73	4.61	3.43	3.5	4.59	1	2.66	1.98	2.02
PCr	4.09	5.21	4.25	4.11	5.13	1	1.27	1.04	1
PCr	5.14	7.61	6.22	5.98	8.14	1	1.48	1.21	1.16
PCr	3.92	5.47	4.46	4.39	5.59	1	1.4	1.14	1.12

	volume(mm3)					volume rate				
CD21	CD7	CD14	CD15	CD16	CD21	CD7	CD14	CD15	CD16	CD21
2.12	0.2	0.32	0.33	0.34	0.43	1	1.56	1.63	1.69	2.12
1.75	0.09	0.12	0.13	0.13	0.16	1	1.38	1.46	1.51	1.75
1.81	0.13	0.19	0.2	0.2	0.24	1	1.45	1.52	1.57	1.81
3.43	0.16	0.43	0.45	0.47	0.56	1	2.64	2.79	2.88	3.43
3.4	0.23	0.6	0.6	0.62	0.77	1	2.63	2.65	2.71	3.4
3.33	0.17	0.44	0.46	0.48	0.58	1	2.52	2.61	2.74	3.33
1.8	0.48	0.71	0.75	0.76	0.86	1	1.47	1.55	1.58	1.8
1.6	0.49	0.67	0.71	0.72	0.78	1	1.38	1.45	1.46	1.6
1.85	0.55	0.82	0.86	0.87	1.01	1	1.51	1.58	1.59	1.85
1.9	0.21	0.33	0.43	0.46	0.64	1	1.57	2.05	2.2	3.04
1.99	0.22	0.35	0.46	0.49	0.71	1	1.58	2.07	2.18	3.19
1.36	0.15	0.23	0.29	0.29	0.33	1	1.49	1.86	1.91	2.17
2.27	0.14	0.34	0.41	0.41	0.52	1	2.37	2.89	2.89	3.64
2.43	0.12	0.3	0.35	0.36	0.47	1	2.48	2.95	2.97	3.88
2.65	0.17	0.46	0.55	0.56	0.73	1	2.66	3.17	3.24	4.25
1.25	0.41	0.52	0.68	0.66	0.82	1	1.27	1.66	1.61	2.01
1.58	0.51	0.76	1	0.96	1.3	1	1.48	1.94	1.86	2.53
1.43	0.39	0.55	0.71	0.7	0.89	1	1.4	1.82	1.79	2.28
