# Supplementary material for: Culture-space control is effective in promoting haploid cell formation and spermiogenesis in vitro in neonatal mice
Source: Sci Rep. 2023 Jul 31;13:12354. doi: 10.1038/s41598-023-39323-y (PMC10390558; doi:10.1038/s41598-023-39323-y)
Supplement: Supplementary file 3 — Supplementary Information 3. [file 41598_2023_39323_MOESM3_ESM.pdf]

Seminiferous tubule diameter(μm) before and after PC-chip replacement

| PC-r#1 | | PC-r#2 | | PC-r#3 | | PC-r#4 | | PC-r#5 | | PC-r#6 | |
|---|---|---|---|---|---|---|---|---|---|---|---|
| CD14 | CD15 | CD14 | CD15 | CD14 | CD15 | CD14 | CD15 | CD14 | CD15 | CD14 | CD15 |
| 122 | 134 | 93 | 99 | 118 | 119 | 116 | 85 | 87 | 88 | 87 | 110 |
| 132 | 100 | 123 | 113 | 105 | 99 | 80 | 71 | 83 | 70 | 115 | 85 |
| 126 | 112 | 127 | 117 | 114 | 111 | 93 | 94 | 97 | 81 | 104 | 124 |
| 114 | 135 | 119 | 115 | 131 | 99 | 87 | 94 | 103 | 89 | 107 | 90 |
| 115 | 90 | 102 | 104 | 114 | 99 | 81 | 86 | 90 | 113 | 94 | 101 |
| 111 | 107 | 112 | 100 | 119 | 92 | 100 | 89 | 81 | 82 | 112 | 99 |
| 121 | 112 | 99 | 101 | 121 | 101 | 86 | 75 | 95 | 96 | 106 | 89 |
| 135 | 113 | 130 | 132 | 154 | 87 | 99 | 79 | 99 | 85 | 111 | 79 |
| 102 | 107 | 110 | 113 | 106 | 108 | 86 | 95 | 104 | 90 | 108 | 96 |

| PC-r#7 | | PC-r#8 | | PC-r#9 | |
|---|---|---|---|---|---|
| CD14 | CD15 | CD14 | CD15 | CD14 | CD15 |
| 91 | 80 | 83 | 81 | 83 | 72 |
| 86 | 86 | 81 | 81 | 86 | 71 |
| 85 | 78 | 84 | 87 | 91 | 84 |
| 77 | 87 | 89 | 79 | 92 | 84 |
| 70 | 62 | 101 | 93 | 91 | 69 |
| 83 | 61 | 103 | 84 | 97 | 77 |
| 83 | 84 | 101 | 86 | 89 | 84 |
| 84 | 72 | 87 | 83 | 97 | 81 |
| 93 | 64 | 88 | 89 | 89 | 79 |
