# Supplementary material for: Culture-space control is effective in promoting haploid cell formation and spermiogenesis in vitro in neonatal mice
Source: Sci Rep. 2023 Jul 31;13:12354. doi: 10.1038/s41598-023-39323-y (PMC10390558; doi:10.1038/s41598-023-39323-y)
Supplement: Supplementary file 4 — Supplementary Information 4. [file 41598_2023_39323_MOESM4_ESM.pdf]

Germ cell differentiation grade for each PC chip

	GD0	GD1	GD2	GD3	GD4	total STs
PC60	2	12	13	0	0	27
PC60	2	34	8	0	0	44
PC60	0	4	9	0	0	13
PC60	7	13	0	0	0	20
PC60	14	17	0	0	0	31
PC60	15	31	9	0	0	55
PC100	4	4	49	0	0	57
PC100	3	14	39	0	0	56
PC100	1	2	30	0	0	33
PC100	3	11	25	2	1	42
PC100	8	28	0	0	0	36
PC100	7	12	30	3	0	52
PC100	6	12	8	0	0	26
PC100	4	1	70	0	0	75
PC160	2	8	129	0	0	139
PC160	0	4	45	3	5	57
PC160	2	9	42	0	0	53
PC160	5	9	42	2	4	62
PC160	4	12	37	4	7	64
PC160	5	19	48	3	2	77
PC160	5	5	60	4	6	80
PC160	5	5	40	6	0	56
PC160	6	1	16	8	7	38
PC-r	0	3	9	34	0	46
PC-r	0	0	14	30	21	65
PC-r	0	0	11	26	4	41
PC-r	8	8	33	4	11	64
PC-r	4	14	76	5	13	112
PC-r	7	13	27	2	1	50
PC-r	6	3	21	3	0	33
PC-r	3	2	43	7	10	65
PC-r	3	3	22	6	6	40
