# Supplementary material for: Culture-space control is effective in promoting haploid cell formation and spermiogenesis in vitro in neonatal mice
Source: Sci Rep. 2023 Jul 31;13:12354. doi: 10.1038/s41598-023-39323-y (PMC10390558; doi:10.1038/s41598-023-39323-y)
Supplement: Supplementary file 5 — Supplementary Information 5. [file 41598_2023_39323_MOESM5_ESM.pdf]

Tissue volume rate for each O_2 concentration

	area(mm^2)					volume(mm^3)				
	CD7	CD14	CD21	CD28	CD35	CD7	CD14	CD21	CD28	CD35
10%	3.30	4.45	4.07	4.03	4.28	0.33	0.45	0.65	0.64	0.68
10%	4.90	6.87	5.35	5.21	5.49	0.49	0.69	0.86	0.83	0.88
10%	3.49	4.75	4.97	5.31	6.20	0.35	0.48	0.80	0.85	0.99
10%	5.54	6.84	3.60	3.26	4.78	0.55	0.68	0.58	0.52	0.76
10%	2.46	2.82	1.56	1.28	1.33	0.25	0.28	0.25	0.20	0.21
10%	4.71	5.19	2.63	2.30	2.98	0.47	0.52	0.42	0.37	0.48
10%	2.37	2.60	1.23	0.86	0.78	0.24	0.26	0.20	0.14	0.12
10%	4.23	4.26	2.74	2.45	3.16	0.42	0.43	0.44	0.39	0.51
10%	3.07	3.44	2.15	2.37	3.45	0.31	0.34	0.34	0.38	0.55
10%	4.15	5.56	3.10	2.56	2.24	0.42	0.56	0.50	0.41	0.36
10%	4.70	7.01	4.10	3.56	3.24	0.47	0.70	0.66	0.57	0.52
10%	3.86	5.96	3.24	2.86	2.59	0.39	0.60	0.52	0.46	0.41
15%	4.53	6.84	7.13	8.42	8.99	0.45	0.68	1.14	1.35	1.44
15%	5.36	9.90	10.74	12.64	13.89	0.54	0.99	1.72	2.02	2.22
15%	5.37	10.11	10.99	11.55	12.06	0.54	1.01	1.76	1.85	1.93
15%	3.87	4.85	2.90	2.92	2.94	0.39	0.49	0.46	0.47	0.47
15%	3.93	4.75	2.79	2.88	2.94	0.39	0.48	0.45	0.46	0.47
15%	3.85	4.16	2.22	2.65	2.87	0.39	0.42	0.36	0.42	0.46
15%	5.88	7.80	6.94	8.08	8.49	0.59	0.78	1.11	1.29	1.36
15%	4.49	4.86	2.52	3.27	3.93	0.45	0.49	0.40	0.52	0.63
15%	4.66	7.83	5.47	4.61	4.61	0.47	0.78	0.88	0.74	0.74
15%	6.31	11.72	8.28	6.28	6.28	0.63	1.17	1.32	1.00	1.00
15%	4.90	8.78	6.26	5.15	5.15	0.49	0.88	1.00	0.82	0.82
20%	4.04	7.49	9.02	10.24	11.45	0.40	0.75	1.44	1.64	1.83
20%	4.67	8.37	9.40	10.58	11.48	0.47	0.84	1.50	1.69	1.84
20%	3.67	7.14	8.59	10.68	11.51	0.37	0.71	1.37	1.71	1.84
20%	4.28	5.32	4.70	6.31	7.38	0.43	0.53	0.75	1.01	1.18
20%	4.43	5.81	4.70	6.34	7.28	0.44	0.58	0.75	1.01	1.16
20%	6.45	7.23	5.93	7.63	8.64	0.65	0.72	0.95	1.22	1.38
20%	4.16	4.22	3.44	4.88	5.14	0.42	0.42	0.55	0.78	0.82
20%	5.35	6.41	5.27	8.14	10.42	0.54	0.64	0.84	1.30	1.67
20%	5.65	7.29	6.84	10.21	10.97	0.57	0.73	1.09	1.63	1.76
20%	4.29	7.26	5.92	5.06	4.55	0.43	0.73	0.95	0.81	0.73
20%	4.51	7.83	6.09	5.41	4.83	0.45	0.78	0.97	0.87	0.77

volume rate

CD7	CD14	CD21	CD28	CD35
1.00	1.35	1.97	1.95	2.08
1.00	1.40	1.75	1.70	1.79
1.00	1.36	2.28	2.43	2.84
1.00	1.23	1.04	0.94	1.38
1.00	1.15	1.01	0.83	0.87
1.00	1.10	0.89	0.78	1.01
1.00	1.10	0.83	0.58	0.53
1.00	1.01	1.04	0.93	1.20
1.00	1.12	1.12	1.24	1.80
1.00	1.34	1.20	0.99	0.86
1.00	1.49	1.40	1.21	1.10
1.00	1.54	1.34	1.19	1.07
1.00	1.51	2.52	2.97	3.18
1.00	1.85	3.21	3.77	4.15
1.00	1.88	3.27	3.44	3.59
1.00	1.25	1.20	1.21	1.22
1.00	1.21	1.14	1.17	1.20
1.00	1.08	0.92	1.10	1.19
1.00	1.33	1.89	2.20	2.31
1.00	1.08	0.90	1.17	1.40
1.00	1.68	1.88	1.58	1.58
1.00	1.86	2.10	1.59	1.59
1.00	1.79	2.04	1.68	1.68
1.00	1.85	3.57	4.06	4.53
1.00	1.79	3.22	3.62	3.93
1.00	1.95	3.74	4.66	5.02
1.00	1.24	1.76	2.36	2.76
1.00	1.31	1.70	2.29	2.63
1.00	1.12	1.47	1.89	2.14
1.00	1.01	1.32	1.88	1.98
1.00	1.20	1.58	2.43	3.12
1.00	1.29	1.94	2.89	3.11
1.00	1.69	2.21	1.89	1.70
1.00	1.74	2.16	1.92	1.71
