# Supplementary material for: Culture-space control is effective in promoting haploid cell formation and spermiogenesis in vitro in neonatal mice
Source: Sci Rep. 2023 Jul 31;13:12354. doi: 10.1038/s41598-023-39323-y (PMC10390558; doi:10.1038/s41598-023-39323-y)
Supplement: Supplementary file 6 — Supplementary Information 6. [file 41598_2023_39323_MOESM6_ESM.pdf]

GFP grade for each O_2 concentration

	CD7	CD14	CD21	CD28	CD35
10%	0	4	5	5	5
10%	0	4	5	5	4
10%	0	3	5	5	5
10%	0	1	4	4	2
10%	0	1	5	5	3
10%	0	3	4	4	1
10%	0	1	3	3	1
10%	0	1	5	5	5
10%	0	1	5	5	4
15%	0	2	5	5	5
15%	0	4	5	5	5
15%	0	3	5	5	5
15%	0	0	5	5	4
15%	0	0	4	4	4
15%	0	0	1	1	1
15%	0	0	5	5	5
15%	0	0	1	1	0
20%	0	2	5	5	5
20%	0	2	5	5	5
20%	0	3	5	5	5
20%	0	0	2	4	2
20%	0	0	2	3	1
20%	0	0	3	4	3
20%	0	0	0	0	0
20%	0	0	3	5	4
20%	0	0	4	4	1
