# Supplementary material for: Culture-space control is effective in promoting haploid cell formation and spermiogenesis in vitro in neonatal mice
Source: Sci Rep. 2023 Jul 31;13:12354. doi: 10.1038/s41598-023-39323-y (PMC10390558; doi:10.1038/s41598-023-39323-y)
Supplement: Supplementary file 7 — Supplementary Information 7. [file 41598_2023_39323_MOESM7_ESM.pdf]

Germ cell differentiation grade (GD) for each O2 concentration

O_2	GD0	GD1	GD2	GD3	GD4	total
20%	0	3	9	34	0	46
20%	0	0	14	30	21	65
20%	0	0	11	26	4	41
20%	3	53	44	5	0	105
20%	2	51	30	24	45	152
20%	8	8	33	4	11	64
20%	4	14	76	5	13	112
20%	7	13	27	2	1	50
20%	6	3	21	3	0	33
20%	3	2	43	7	10	65
20%	3	3	22	6	6	40
15%	7	1	10	17	32	67
15%	2	1	2	3	16	24
15%	17	3	11	8	41	80
15%	6	4	8	8	59	85
15%	4	4	4	5	45	62
15%	23	7	19	32	30	111
15%	0	5	3	12	40	60
15%	0	8	3	9	19	39
15%	0	4	12	53	0	69
15%	2	34	43	14	19	112
15%	1	20	18	19	14	72
15%	5	60	13	1	0	79
10%	27	4	16	4	2	53
10%	35	2	9	3	5	54
10%	21	0	6	5	5	37
10%	23	0	3	5	16	47
10%	42	3	4	3	2	54
10%	26	3	5	1	1	36
10%	7	12	29	3	5	56
10%	19	30	18	0	1	68
10%	49	28	19	0	0	96
