# Supplementary material for: Culture-space control is effective in promoting haploid cell formation and spermiogenesis in vitro in neonatal mice
Source: Sci Rep. 2023 Jul 31;13:12354. doi: 10.1038/s41598-023-39323-y (PMC10390558; doi:10.1038/s41598-023-39323-y)
Supplement: Supplementary file 8 — Supplementary Information 8. [file 41598_2023_39323_MOESM8_ESM.pdf]

Tissue volume rate in EE toxicity test

	area(mm^2)					volume(mm^3)			
	CD7	CD14	CD21	CD28	CD35	CD7	CD14	CD21	CD28
control	5.52	7.85	4.22	5.46		0.55	0.79	0.68	0.87
control	4.12	5.78	3.99	6.33		0.41	0.58	0.64	1.01
control	5.35	7.70	5.91	9.11		0.54	0.77	0.95	1.46
control	6.82	10.37	10.07	12.37	12.10	0.68	1.04	1.61	1.98
control	6.49	9.55	7.50	8.21	8.15	0.65	0.96	1.20	1.31
control	6.80	10.64	7.98	9.23	10.31	0.68	1.06	1.28	1.48
control	5.85	8.41	6.69	8.70	9.55	0.59	0.84	1.07	1.39
control	5.09	7.20	5.86	6.03	5.98	0.51	0.72	0.94	0.96
control	6.66	10.00	9.27	9.61	9.62	0.67	1.00	1.48	1.54
control	4.52	6.91	5.86	6.19	6.64	0.45	0.69	0.94	0.99
control	4.51	6.70	6.38	6.52	6.71	0.45	0.67	1.02	1.04
control	3.29	5.00	5.54	5.76	5.95	0.33	0.50	0.89	0.92
control	6.13	9.70	10.91	13.32	15.70	0.61	0.97	1.75	2.13
control	5.57	8.26	8.35	9.72	10.62	0.56	0.83	1.34	1.56
control	6.98	10.85	10.35	13.25	15.44	0.70	1.09	1.66	2.12
control	5.53	7.45	10.05	9.56	9.68	0.55	0.75	1.61	1.53
control	4.98	6.83	9.16	8.71	8.54	0.50	0.68	1.47	1.39
control	6.79	8.89	11.98	11.78	12.28	0.68	0.89	1.92	1.88
EE0.01	3.37	4.23	4.18	5.44	5.64	0.34	0.42	0.67	0.87
EE0.01	5.03	6.93	5.92	7.61	9.15	0.50	0.69	0.95	1.22
EE0.01	4.86	6.65	5.32	5.50	5.55	0.49	0.67	0.85	0.88
EE0.01	3.96	5.08	4.61	4.91	4.78	0.40	0.51	0.74	0.79
EE0.01	3.94	5.32	4.18	4.23	4.31	0.39	0.53	0.67	0.68
EE0.01	4.43	5.32	7.07	6.63	6.40	0.44	0.53	1.13	1.06
EE0.01	5.20	6.48	6.91	6.71	8.35	0.52	0.65	1.11	1.07
EE0.01	4.59	5.41	6.78	6.63	6.61	0.46	0.54	1.08	1.06
EE0.1	6.61	9.61	9.52	10.04	9.81	0.66	0.96	1.52	1.61
EE0.1	3.52	4.70	4.07	3.84	3.44	0.35	0.47	0.65	0.61
EE0.1	4.10	5.46	4.52	4.78	4.86	0.41	0.55	0.72	0.76
EE0.1	4.65	5.73	3.79	3.75		0.47	0.57	0.61	0.60
EE0.1	6.79	8.38	5.73	6.04		0.68	0.84	0.92	0.97
EE0.1	3.96	4.43	2.18	1.74		0.40	0.44	0.35	0.28
EE0.1	6.04	8.98	7.12	8.51	6.56	0.60	0.90	1.14	1.36
EE0.1	6.24	9.10	5.86	5.17	4.39	0.62	0.91	0.94	0.83
EE0.1	5.45	8.04	6.17	6.37	5.54	0.55	0.80	0.99	1.02
EE0.1	5.87	9.07	7.84	9.91	8.82	0.59	0.91	1.25	1.59

EE0.1	4.69	6.94	4.47	3.88	3.39	0.47	0.69	0.72	0.62
EE0.1	7.43	10.87	7.56	7.31	6.36	0.74	1.09	1.21	1.17
EE0.1	5.16	6.68	4.86	5.17	5.11	0.52	0.67	0.78	0.83
EE0.1	3.25	3.95	3.74	3.89	3.59	0.33	0.40	0.60	0.62
EE0.1	4.07	5.07	4.58	5.13	4.91	0.41	0.51	0.73	0.82
EE1.0	5.02	7.10	6.39	5.62	4.99	0.50	0.71	1.02	0.90
EE1.0	5.68	7.24	5.75	4.75	4.04	0.57	0.72	0.92	0.76
EE1.0	3.79	4.51	3.65	3.28	2.85	0.38	0.45	0.58	0.52
EE1.0	4.73	6.05	3.94	3.77		0.47	0.61	0.63	0.60
EE1.0	5.24	6.31	4.13	3.93		0.52	0.63	0.66	0.63
EE1.0	5.23	7.12	5.92	5.98	5.26	0.52	0.71	0.95	0.96
EE1.0	6.35	9.05	6.00	5.45	4.54	0.64	0.91	0.96	0.87
EE1.0	7.22	9.99	7.22	6.60	5.92	0.72	1.00	1.16	1.06
EE1.0	5.49	7.59	5.04	5.23	4.50	0.55	0.76	0.81	0.84
EE1.0	5.50	7.97	5.87	5.34	4.56	0.55	0.80	0.94	0.85
EE1.0	6.64	9.62	6.55	6.08	5.33	0.66	0.96	1.05	0.97
EE1.0	4.46	5.70	4.52	4.90	4.64	0.45	0.57	0.72	0.78
EE1.0	4.64	6.05	4.43	4.42	4.29	0.46	0.61	0.71	0.71
EE1.0	4.98	7.14	4.89	5.31	5.40	0.50	0.71	0.78	0.85

	volume rate				
CD35	CD7	CD14	CD21	CD28	CD35
	1.00	1.42	1.22	1.58	
	1.00	1.40	1.55	2.46	
	1.00	1.44	1.77	2.72	
1.94	1.00	1.52	2.36	2.90	2.84
1.30	1.00	1.47	1.85	2.02	2.01
1.65	1.00	1.56	1.88	2.17	2.43
1.53	1.00	1.44	1.83	2.38	2.61
0.96	1.00	1.41	1.84	1.90	1.88
1.54	1.00	1.50	2.23	2.31	2.31
1.06	1.00	1.53	2.07	2.19	2.35
1.07	1.00	1.49	2.26	2.31	2.38
0.95	1.00	1.52	2.69	2.80	2.89
2.51	1.00	1.58	2.85	3.48	4.10
1.70	1.00	1.48	2.40	2.79	3.05
2.47	1.00	1.55	2.37	3.04	3.54
1.55	1.00	1.35	2.91	2.77	2.80
1.37	1.00	1.37	2.94	2.80	2.74
1.96	1.00	1.31	2.82	2.78	2.89
0.90	1.00	1.26	1.98	2.58	2.68
1.46	1.00	1.38	1.88	2.42	2.91
0.89	1.00	1.37	1.75	1.81	1.83
0.76	1.00	1.28	1.86	1.98	1.93
0.69	1.00	1.35	1.70	1.72	1.75
1.02	1.00	1.20	2.55	2.39	2.31
1.34	1.00	1.25	2.13	2.06	2.57
1.06	1.00	1.18	2.36	2.31	2.30
1.57	1.00	1.45	2.30	2.43	2.37
0.55	1.00	1.34	1.85	1.75	1.56
0.78	1.00	1.33	1.76	1.87	1.90
	1.00	1.23	1.30	1.29	
	1.00	1.23	1.35	1.42	
	1.00	1.12	0.88	0.70	
1.05	1.00	1.49	1.89	2.25	1.74
0.70	1.00	1.46	1.50	1.33	1.13
0.89	1.00	1.48	1.81	1.87	1.63
1.41	1.00	1.55	2.14	2.70	2.40

0.54	1.00	1.48	1.52	1.32	1.16
1.02	1.00	1.46	1.63	1.57	1.37
0.82	1.00	1.29	1.51	1.60	1.58
0.57	1.00	1.22	1.84	1.92	1.77
0.79	1.00	1.25	1.80	2.02	1.93
0.80	1.00	1.41	2.04	1.79	1.59
0.65	1.00	1.27	1.62	1.34	1.14
0.46	1.00	1.19	1.54	1.38	1.20
	1.00	1.28	1.33	1.28	
	1.00	1.20	1.26	1.20	
0.84	1.00	1.36	1.81	1.83	1.61
0.73	1.00	1.43	1.51	1.37	1.14
0.95	1.00	1.38	1.60	1.46	1.31
0.72	1.00	1.38	1.47	1.52	1.31
0.73	1.00	1.45	1.71	1.55	1.33
0.85	1.00	1.45	1.58	1.47	1.28
0.74	1.00	1.28	1.62	1.76	1.66
0.69	1.00	1.30	1.53	1.52	1.48
0.86	1.00	1.43	1.57	1.71	1.73
