# Supplementary material for: Culture-space control is effective in promoting haploid cell formation and spermiogenesis in vitro in neonatal mice
Source: Sci Rep. 2023 Jul 31;13:12354. doi: 10.1038/s41598-023-39323-y (PMC10390558; doi:10.1038/s41598-023-39323-y)
Supplement: Supplementary file 9 — Supplementary Information 9. [file 41598_2023_39323_MOESM9_ESM.pdf]

seminiferous tubule diameter (μm) in EE toxicity test

	CD14	CD28		CD14	CD28
control	83.3	87.6	EE0.1	84.0	67.5
control	68.3	67.8	EE0.1	83.4	71.7
control	67.5	76.2	EE0.1	88.0	90.8
control	81.0	76.2	EE0.1	77.4	60.0
control	68.3	55.8	EE0.1	78.8	63.3
control	65.3	59.4	EE0.1	90.7	72.5
control	62.3	75.6	EE0.1	60.2	63.3
control	68.3	64.2	EE0.1	86.7	66.7
control	78.0	68.4	EE0.1	86.7	84.2
control	89.3	87.6	EE0.1	89.3	71.7
control	80.3	72.0	EE0.1	62.9	65.8
control	78.8	67.2	EE0.1	75.4	79.2
control	73.5	83.4	EE0.1	80.7	55.8
control	54.8	63.6	EE0.1	72.1	76.7
control	61.5	56.4	EE0.1	92.6	50.8
control	56.3	63.0	EE0.1	72.1	58.3
control	56.3	72.0	EE0.1	70.1	62.5
control	73.5	85.2	EE0.1	72.1	74.2
control	75.8	66.6	EE0.1	84.7	65.8
control	68.3	63.6	EE0.1	69.5	68.3
control	69.8	75.6	EE0.1	71.5	71.7
control	83.3	68.4	EE0.1	78.1	78.3
control	54.8	60.0	EE0.1	76.8	77.5
control	71.3	71.4	EE0.1	73.5	73.3
control	59.3	71.4	EE0.1	83.0	86.0
control	60.0	79.8	EE0.1	104.0	96.0
control	62.3	65.4	EE0.1	113.0	68.0
control	107.0	105.0	EE0.1	96.0	66.0
control	107.0	112.0	EE0.1	103.0	106.0
control	127.0	145.0	EE0.1	95.0	82.0
control	113.0	102.0	EE0.1	101.0	95.0
control	110.0	119.0	EE0.1	108.0	90.0
control	113.0	125.0	EE0.1	110.0	88.0
control	116.0	106.0	EE0.1	92.0	77.0
control	108.0	123.0	EE0.1	109.0	88.0
control	105.0	156.0	EE0.1	90.0	77.0
control	116.0	90.0	EE0.1	111.0	107.0

control	104.0	132.0	EE0.1	113.0	86.0
control	113.0	136.0	EE0.1	88.0	91.0
control	123.0	95.0	EE0.1	93.0	96.0
control	106.0	137.0	EE0.1	116.0	110.0
control	102.0	133.0	EE0.1	77.0	82.0
control	118.0	97.0	EE0.1	92.0	87.0
control	119.0	112.0	EE0.1	103.0	90.0
control	122.0	136.0	EE0.1	77.0	71.0
control	131.0	104.0	EE0.1	96.0	92.0
control	116.0	122.0	EE0.1	113.0	100.0
control	106.0	129.0	EE0.1	94.0	64.0
control	125.0	88.0	EE0.1	72.0	70.0
control	126.0	132.0	EE0.1	91.0	73.0
control	114.0	134.0	EE0.1	72.0	63.0
control	83.0	83.0	EE0.1	73.0	79.5
control	78.0	132.0	EE0.1	74.5	101.3
control	87.0	115.0	EE0.1	74.5	87.8
control	73.5	105.0	EE0.1	69.2	72.0
control	66.8	94.5	EE0.1	75.3	63.0
control	70.5	104.3	EE0.1	66.2	75.8
control	75.8	104.3	EE0.1	76.0	81.8
control	68.3	102.0	EE0.1	69.2	57.8
control	92.3	107.3	EE0.1	76.8	78.0
control	68.3	107.3	EE0.1	67.5	56.3
control	66.8	102.0	EE0.1	56.3	67.5
control	84.0	94.5	EE0.1	56.3	52.5
control	73.5	104.3	EE0.1	72.0	57.8
control	72.0	88.5	EE0.1	61.5	58.5
control	71.3	105.0	EE0.1	68.3	64.5
control	67.5	102.8	EE0.1	52.5	61.5
control	57.8	97.5	EE0.1	63.0	53.3
control	72.8	89.3	EE0.1	65.3	74.3
control	70.5	80.3	EE0.1	73.5	60.0
control	73.5	91.5	EE0.1	77.3	83.3
control	69.8	93.0	EE0.1	71.3	75.8
control	67.7	111.8	EE0.1	66.8	69.8
control	91.8	90.0	EE0.1	60.8	69.0
control	68.5	81.0	EE0.1	70.5	69.0
control	71.5	105.0	EE0.1	73.5	75.0

control	54.9	80.3	EE0.1	63.0	63.0
control	62.5	99.8	EE0.1	61.5	57.8
control	68.5	84.0			
control	61.7	100.5			
control	66.2	90.8			
EE0.01	71.3	73.2	EE1.0	50.3	63.3
EE0.01	69.0	70.8	EE1.0	35.7	45.0
EE0.01	66.8	70.2	EE1.0	47.6	60.0
EE0.01	67.5	63.0	EE1.0	63.5	80.0
EE0.01	59.3	64.2	EE1.0	35.7	45.0
EE0.01	69.8	56.4	EE1.0	54.9	69.2
EE0.01	75.0	60.0	EE1.0	61.5	77.5
EE0.01	68.3	66.6	EE1.0	48.3	60.8
EE0.01	68.3	77.4	EE1.0	56.3	70.8
EE0.01	66.8	75.6	EE1.0	62.9	79.2
EE0.01	69.0	72.6	EE1.0	56.3	70.8
EE0.01	73.5	102.0	EE1.0	48.3	60.8
EE0.01	68.3	70.2	EE1.0	63.5	80.0
EE0.01	63.8	77.4	EE1.0	53.6	67.5
EE0.01	58.5	74.4	EE1.0	59.6	75.0
EE0.01	60.8	72.0	EE1.0	45.7	57.5
EE0.01	75.8	75.0	EE1.0	49.0	61.7
EE0.01	64.5	79.8	EE1.0	62.2	78.3
EE0.01	73.5	82.2	EE1.0	48.3	60.8
EE0.01	69.8	61.2	EE1.0	45.7	57.5
EE0.01	63.8	65.4	EE1.0	52.9	66.7
EE0.01	67.5	77.4	EE1.0	64.9	81.7
EE0.01	51.8	64.8	EE1.0	51.0	64.2
EE0.01	69.0	65.4	EE1.0	45.7	57.5
EE0.01	69.0	76.8	EE1.0	105.0	103.0
EE0.01	66.8	67.8	EE1.0	107.0	102.0
EE0.01	66.8	67.2	EE1.0	88.0	
EE0.01	74.5	71.4	EE1.0	109.0	88.0
EE0.01	62.6	89.6	EE1.0	91.0	84.0
EE0.01	81.7	90.4	EE1.0	102.0	
EE0.01	81.7	87.2	EE1.0	110.0	87.0
EE0.01	80.9	90.4	EE1.0	102.0	85.0
EE0.01	76.9	88.0	EE1.0	82.0	

EE0.01	76.9	82.5	EE1.0	112.0	97.0
EE0.01	87.2	70.6	EE1.0	86.0	87.0
EE0.01	69.8	92.0	EE1.0	126.0	
EE0.01	92.8	87.2	EE1.0	105.0	87.0
EE0.01	80.9	79.3	EE1.0	105.0	68.0
EE0.01	84.8	84.1	EE1.0	90.0	
EE0.01	77.7	73.7	EE1.0	107.0	80.0
EE0.01	71.4	88.0	EE1.0	107.0	101.0
EE0.01	66.6	88.8	EE1.0	75.0	
EE0.01	68.2	79.3	EE1.0	105.0	81.0
EE0.01	69.0	90.4	EE1.0	98.0	102.0
EE0.01	84.1	92.8	EE1.0	99.0	
EE0.01	71.4	88.0	EE1.0	92.0	114.0
EE0.01	80.1	93.6	EE1.0	89.0	84.0
EE0.01	83.3	90.4	EE1.0	98.0	
EE0.01	78.5	86.4	EE1.0	67.0	64.0
EE0.01	74.5	82.5	EE1.0	73.0	77.0
EE0.01	65.0	78.5	EE1.0	74.0	72.0
EE0.01	69.8	73.0	EE1.0	66.0	41.3
EE0.01	88.0	92.8	EE1.0	65.3	57.0
EE0.01	84.1	68.2	EE1.0	64.5	59.3
EE0.01	70.6	76.1	EE1.0	61.5	81.0
EE0.01	72.2	78.5	EE1.0	52.5	56.3
EE0.01	70.6	77.7	EE1.0	70.5	56.3
EE0.01	80.1	71.4	EE1.0	60.0	68.3
EE0.01	72.2	86.4	EE1.0	60.8	54.8
EE0.01	69.8	84.1	EE1.0	69.8	63.0
EE0.01	88.8	81.7	EE1.0	69.8	56.3
EE0.01	78.5	76.1	EE1.0	60.8	60.8
EE0.01	86.4	76.1	EE1.0	60.8	66.8
EE0.01	63.0	82.5	EE1.0	65.3	63.0
EE0.01	64.5	78.0	EE1.0	55.5	53.3
EE0.01	56.3	72.8	EE1.0	59.3	57.0
EE0.01	65.3	78.8	EE1.0	61.5	63.8
EE0.01	51.8	66.8	EE1.0	70.5	70.5
EE0.01	61.5	74.3	EE1.0	69.0	57.0
EE0.01	66.8	86.3	EE1.0	57.0	41.3
EE0.01	62.3	87.0	EE1.0	66.0	60.0
EE0.01	68.3	87.8	EE1.0	70.5	62.3

EE0.01	65.3	85.5	EE1.0	63.8	65.3
EE0.01	59.3	84.8	EE1.0	58.5	57.8
EE0.01	63.0	81.8	EE1.0	63.8	54.8
EE0.01	76.5	68.3	EE1.0	54.0	66.0
EE0.01	53.3	76.5	EE1.0	63.0	69.0
EE0.01	58.5	78.8	EE1.0	51.0	65.3
EE0.01	75.0	68.3			
EE0.01	63.0	90.8			
EE0.01	69.0	77.3			
