# Supplementary material for: Culture-space control is effective in promoting haploid cell formation and spermiogenesis in vitro in neonatal mice
Source: Sci Rep. 2023 Jul 31;13:12354. doi: 10.1038/s41598-023-39323-y (PMC10390558; doi:10.1038/s41598-023-39323-y)
Supplement: Supplementary file 10 — Supplementary Information 10. [file 41598_2023_39323_MOESM10_ESM.pdf]

GFP grade in EE toxicity test

	CD7	CD14	CD21	CD28	CD35
control	0	5	2	4	
control	0	4	2	4	
control	0	4	4	1	
control	0	1	5	5	4
control	0	1	5	5	4
control	0	1	5	5	3
control	0	1	5	4	4
control	0	1	5	4	4
control	0	2	5	4	2
control	0	1	4	4	5
control	0	3	5	5	5
control	0	1	5	5	5
control	0	5	5	5	5
control	0	5	4	4	4
control	0	5	5	5	5
control	0	1	5	5	4
control	0	1	5	5	5
control	0	1	5	5	5
EE0.01	0	5	5	5	5
EE0.01	0	5	4	4	4
EE0.01	0	1	4	4	5
EE0.01	0	2	4	4	5
EE0.01	0	1	4	5	5
EE0.01	0	1	5	4	4
EE0.01	0	1	4	5	5
EE0.01	0	1	3	3	3
EE0.1	0	5	5	5	5
EE0.1	0	4	5	5	5
EE0.1	0	5	5	4	4
EE0.1	0	4	2	3	
EE0.1	0	5	4	5	
EE0.1	0	1	1	1	
EE0.1	0	1	5	4	5
EE0.1	0	1	5	5	5
EE0.1	0	1	5	5	5

EE0.1	0	1	5	4	4
EE0.1	0	1	5	5	5
EE0.1		2	5	5	5
EE0.1	0	0	2	2	4
EE0.1	0	1	4	4	4
EE0.1	0	0	3	4	5

EE1.0	0	5	5	5
EE1.0	0	3	4	4
EE1.0	0	5	5	2
EE1.0	0	4	4	4
EE1.0	0	4	4	4
EE1.0	0	1	5	5
EE1.0	0	1	5	5
EE1.0	0	2	5	5
EE1.0	0	2	5	5
EE1.0	0	2	5	5
EE1.0	0	3	5	5
EE1.0	0	1	4	4
EE1.0	0	1	4	4
EE1.0	0	0	3	3
