# Supplementary material for: Culture-space control is effective in promoting haploid cell formation and spermiogenesis in vitro in neonatal mice
Source: Sci Rep. 2023 Jul 31;13:12354. doi: 10.1038/s41598-023-39323-y (PMC10390558; doi:10.1038/s41598-023-39323-y)
Supplement: Supplementary file 11 — Supplementary Information 11. [file 41598_2023_39323_MOESM11_ESM.pdf]

	total STs	ST wtih PaGC	ST with RS	RS	%ST(P)	%ST(RS)	RS/STs
control	90	86	36	385	95.6%	40.0%	4.28
control	106	83	30	113	78.3%	28.3%	1.07
control	224	197	79	393	87.9%	35.3%	1.75
control	315	285	65	298	90.5%	20.6%	0.95
control	123	108	5	41	87.8%	4.1%	0.33
EE0.01	84	72	17	26	85.7%	20.2%	0.31
EE0.01	96	87	19	39	90.6%	19.8%	0.41
EE0.01	111	97	12	24	87.4%	10.8%	0.22
EE0.01	147	135	21	126	91.8%	14.3%	0.86
EE0.01	84	62	15	62	73.8%	17.9%	0.74
EE0.1	89	70	5	10	78.7%	5.6%	0.11
EE0.1	93	77	10	15	82.8%	10.8%	0.16
EE0.1	147	137	10	21	93.2%	6.8%	0.14
EE0.1	116	104	5	24	89.7%	4.3%	0.21
EE0.1	105	92	2	3	87.6%	1.9%	0.03
EE1.0	50	44	3	3	88.0%	6.0%	0.06
EE1.0	76	65	1	1	85.5%	1.3%	0.01
EE1.0	91	84	2	4	92.3%	2.2%	0.04
EE1.0	129	111	1	1	86.0%	0.8%	0.01
EE1.0	136	119	1	1	87.5%	0.7%	0.01
