# Supplementary material for: Culture-space control is effective in promoting haploid cell formation and spermiogenesis in vitro in neonatal mice
Source: Sci Rep. 2023 Jul 31;13:12354. doi: 10.1038/s41598-023-39323-y (PMC10390558; doi:10.1038/s41598-023-39323-y)
Supplement: Supplementary file 12 — Supplementary Information 12. [file 41598_2023_39323_MOESM12_ESM.pdf]

Change of the tissue for each PC-chip

	area(mm2)					volume(mm3)					volume rate				
	CD7	CD14	CD21	CD28	CD35	CD7	CD14	CD21	CD28	CD35	CD7	CD14	CD21	CD28	CD35
PC60	1.5	1.98	2.53	3.33	3.71	0.09	0.12	0.15	0.2	0.22	1	1.32	1.69	2.22	2.47
PC60	3.54	4.82	6.11	7.48	8.44	0.21	0.29	0.37	0.45	0.51	1	1.36	1.73	2.11	2.38
PC60	2.71	3.35	4.24	5.32	5.91	0.16	0.2	0.25	0.32	0.35	1	1.24	1.56	1.96	2.18
PC60	2.69	3.86	4.96	5.73	6.2	0.16	0.23	0.3	0.34	0.37	1	1.43	1.84	2.13	2.3
PC60	1.75	2.93	4.2	5.47		0.11	0.18	0.25	0.33		1	1.67	2.4	3.13	
PC60	1.77	2.61	3.88	4.17	4.49	0.11	0.16	0.23	0.25	0.27	1	1.47	2.19	2.36	2.54
PC60	2.31	3.8	5.43	6.18	6.68	0.14	0.23	0.33	0.37	0.4	1	1.65	2.35	2.68	2.89
PC60	3.16	5.38	7.47	8.72	9.36	0.19	0.32	0.45	0.52	0.56	1	1.7	2.36	2.76	2.96
PC60	1.21	1.08	1.3	1.42	1.42	0.07	0.06	0.08	0.09	0.09	1	0.89	1.07	1.17	1.17
PC60	1.61	1.72	2	2.17	2.08	0.1	0.1	0.12	0.13	0.12	1	1.07	1.24	1.35	1.29
PC60	3.84	5.34	6.79	7.07	7.09	0.23	0.32	0.41	0.42	0.43	1	1.39	1.77	1.84	1.85
PC60	5.17	6.75	8.19	8.68	8.87	0.31	0.41	0.49	0.52	0.53	1	1.31	1.58	1.68	1.72
PC60	4.47	6.74	9.14	9.7	9.75	0.27	0.4	0.55	0.58	0.59	1	1.51	2.04	2.17	2.18
PC60	3.91	5.78	6.79	7.34	8.32	0.23	0.35	0.41	0.44	0.5	1	1.48	1.74	1.88	2.13
PC60	3.68	5.11	5.88	6.69	7.73	0.22	0.31	0.35	0.4	0.46	1	1.39	1.6	1.82	2.1
PC60	3.28	4.93	6.16	6.61	7.39	0.2	0.3	0.37	0.4	0.44	1	1.5	1.88	2.02	2.25
PC60	3.49	5.47	6.78	8.08	10.1	0.21	0.33	0.41	0.48	0.61	1	1.57	1.94	2.32	2.9
PC60	2.87	4.03	4.81	5.96	6.83	0.17	0.24	0.29	0.36	0.41	1	1.4	1.68	2.08	2.38
PC60	2.13	3.03	3.53	4.07	4.3	0.13	0.18	0.21	0.24	0.26	1	1.42	1.66	1.91	2.02
PC100	2.08	3.06	4.01	4.37	4.72	0.21	0.31	0.4	0.44	0.47	1	1.47	1.93	2.1	2.27
PC100	0.84	1.02	1.41	1.53	1.52	0.08	0.1	0.14	0.15	0.15	1	1.21	1.68	1.82	1.81
PC100	0.86	0.97	1.32	1.64	1.73	0.09	0.1	0.13	0.16	0.17	1	1.13	1.53	1.91	2.01
PC100	0.86	1.02	1.43	2.09	2.05	0.09	0.1	0.14	0.21	0.21	1	1.19	1.66	2.43	2.38
PC100	1.11	1.41	1.69	2.01	2.23	0.11	0.14	0.17	0.2	0.22	1	1.27	1.52	1.81	2.01
PC100	2.88	4.51	5.86	6.02	6.33	0.29	0.45	0.59	0.6	0.63	1	1.57	2.03	2.09	2.2
PC100	1.1	2.7	3.96	4.31	4.44	0.11	0.27	0.4	0.43	0.44	1	2.45	3.6	3.92	4.04
PC100	1.74	2.91	4.08	4.86	5.19	0.17	0.29	0.41	0.49	0.52	1	1.67	2.34	2.79	2.98
PC100	1.72	3.07	4.32	5.16	5.62	0.17	0.31	0.43	0.52	0.56	1	1.78	2.51	3	3.27
PC100	0.64	0.64	0.85	0.99	1.04	0.06	0.06	0.09	0.1	0.1	1	1	1.33	1.55	1.63
PC100	0.74	0.74	1	1.16	1.21	0.07	0.07	0.1	0.12	0.12	1	1	1.35	1.57	1.64
PC100	3.28	4.35	5.9	6.42	6.42	0.33	0.44	0.59	0.64	0.64	1	1.33	1.8	1.96	1.96
PC100	2.54	3.49	4.2	4.77	5.5	0.25	0.35	0.42	0.48	0.55	1	1.37	1.65	1.88	2.17
PC100	2.1	2.7	3.7	4.03	4.24	0.21	0.27	0.37	0.4	0.42	1	1.29	1.76	1.92	2.02
PC100	2.02	3.16	4.28	5.08	5.88	0.2	0.32	0.43	0.51	0.59	1	1.56	2.12	2.51	2.91
PC100	0.89	1.23	1.56	1.79	2.12	0.09	0.12	0.16	0.18	0.21	1	1.38	1.75	2.01	2.38
PC100	1.3	1.88	2.35	2.65	3.18	0.13	0.19	0.24	0.27	0.32	1	1.45	1.81	2.04	2.45

PC100	1.62	2.69	3.49	4.4	5.08	0.16	0.27	0.35	0.44	0.51	1	1.66	2.15	2.72	3.14
PC100	2.28	3.75	4.86	6.4	7.69	0.23	0.38	0.49	0.64	0.77	1	1.64	2.13	2.81	3.37
PC100	1.74	2.76	3.64	4.74	5.62	0.17	0.28	0.36	0.47	0.56	1	1.59	2.09	2.72	3.23
PC160	0.76	0.96	1.24	1.23	1.2	0.12	0.15	0.2	0.2	0.19	1	1.26	1.63	1.62	1.58
PC160	0.66	0.93	1.1	1.15	1.21	0.11	0.15	0.18	0.18	0.19	1	1.41	1.67	1.74	1.83
PC160	0.8	1.05	1.45	1.5	1.45	0.13	0.17	0.23	0.24	0.23	1	1.31	1.81	1.88	1.81
PC160	0.67	0.87	1.13	1.32	1.45	0.11	0.14	0.18	0.21	0.23	1	1.3	1.69	1.97	2.16
PC160	0.84	1	1.2	1.26	1.31	0.13	0.16	0.19	0.2	0.21	1	1.19	1.43	1.5	1.56
PC160	1.06	1.37	1.65	1.82	2.04	0.17	0.22	0.26	0.29	0.33	1	1.29	1.56	1.72	1.92
PC160	0.84	1.2	1.88	2.2	2.24	0.13	0.19	0.3	0.35	0.36	1	1.43	2.24	2.62	2.67
PC160	0.87	1.49	2.05	2.44	2.56	0.14	0.24	0.33	0.39	0.41	1	1.71	2.36	2.8	2.94
PC160	0.74	1.13	1.55	1.82	2.01	0.12	0.18	0.25	0.29	0.32	1	1.53	2.09	2.46	2.72
PC160	1.27	1.21	1.35	1.4	1.46	0.2	0.19	0.22	0.22	0.23	1	0.95	1.06	1.1	1.15
PC160	0.78	0.72	0.94	1.05	1.01	0.12	0.12	0.15	0.17	0.16	1	0.92	1.21	1.35	1.29
PC160	1.92	2.39	2.85	3.19	3.23	0.31	0.38	0.46	0.51	0.52	1	1.24	1.48	1.66	1.68
PC160	1.48	2.06	2.54	2.83	3.05	0.24	0.33	0.41	0.45	0.49	1	1.39	1.72	1.91	2.06
PC160	2.02	2.66	3.51	3.55	3.34	0.32	0.43	0.56	0.57	0.53	1	1.32	1.74	1.76	1.65
PC160	1.36	2.07	3.47	5.06	6.12	0.22	0.33	0.56	0.81	0.98	1	1.52	2.55	3.72	4.5
PC160	1.29	1.91	2.39	2.41	2.42	0.21	0.31	0.38	0.39	0.39	1	1.48	1.85	1.87	1.88
PC160	1.36	2.08	3.01	3.79	4.27	0.22	0.33	0.48	0.61	0.68	1	1.53	2.21	2.79	3.14
PC160	1.27	1.94	2.59	3.49	4.32	0.2	0.31	0.41	0.56	0.69	1	1.53	2.04	2.75	3.4
PC160	1.24	1.78	2.22	2.88	3.89	0.2	0.28	0.36	0.46	0.62	1	1.44	1.79	2.32	3.14
PC160	1	1.6	2.12	3.27	4.01	0.16	0.26	0.34	0.52	0.64	1	1.6	2.12	3.27	4.01
PC-r	1.37	1.94	2.05	2.65	3.1	0.14	0.19	0.33	0.42	0.5	1	1.42	2.39	3.09	3.62
PC-r	1.09	1.84	2.29	3.39	3.28	0.11	0.18	0.37	0.54	0.52	1	1.69	3.36	4.98	4.81
PC-r	1.32	2.12	2.58	3.11	3.28	0.13	0.21	0.41	0.5	0.52	1	1.61	3.13	3.77	3.98
PC-r	1.71	1.84	1.72	2.42	2.85	0.17	0.18	0.28	0.39	0.46	1	1.08	1.61	2.26	2.67
PC-r	0.87	0.76	0.88	0.75	0.83	0.09	0.08	0.14	0.12	0.13	1	0.87	1.62	1.38	1.53
PC-r	0.55	0.83	0.8	1.06	1.35	0.06	0.08	0.13	0.17	0.22	1	1.51	2.33	3.08	3.93
PC-r	0.93	1.51	1.55	1.9	2.22	0.09	0.15	0.25	0.3	0.36	1	1.62	2.67	3.27	3.82
PC-r	0.8	1.14	1.03	1.41	1.8	0.08	0.11	0.16	0.23	0.29	1	1.43	2.06	2.82	3.6
PC-r	0.85	1.27	1.28	1.47	1.76	0.09	0.13	0.2	0.24	0.28	1	1.49	2.41	2.77	3.31
PC-r	1.11	1.67	1.67	2.01	2.3	0.11	0.17	0.27	0.32	0.37	1	1.5	2.41	2.9	3.32
PC-r	1.2	1.82	2.03	2.64	3.31	0.12	0.18	0.32	0.42	0.53	1	1.52	2.71	3.52	4.41
PC-r	2.08	2.39	2.47	2.87	3.11	0.21	0.24	0.4	0.46	0.5	1	1.15	1.9	2.21	2.39
PC-r	2.22	2.89	3	3.57	3.93	0.22	0.29	0.48	0.57	0.63	1	1.3	2.16	2.57	2.83
PC-r	1.77	2.29	2.63	3.1	3.54	0.18	0.23	0.42	0.5	0.57	1	1.29	2.38	2.8	3.2
PC-r	1.75	2.45	2.15	2.61	2.75	0.18	0.25	0.34	0.42	0.44	1	1.4	1.97	2.39	2.51
PC-r	2.23	3.09	2.98	3.53	4.19	0.22	0.31	0.48	0.56	0.67	1	1.39	2.14	2.53	3.01

PC-r	2	2.78	2.31	2.5	2.67	0.2	0.28	0.37	0.4	0.43	1	1.39	1.85	2	2.14
PC-r	2.08	2.87	2.53	2.66	2.86	0.21	0.29	0.4	0.43	0.46	1	1.38	1.95	2.05	2.2
PC-r	2.32	3.26	3.2	4.19	4.17	0.23	0.33	0.51	0.67	0.67	1	1.41	2.21	2.89	2.88
PC-r	2.6	3.71	3.55	4.18	5.11	0.26	0.37	0.57	0.67	0.82	1	1.43	2.18	2.57	3.14
PC-r	2.11	3.31	4.01	5.05	5.74	0.21	0.33	0.64	0.81	0.92	1	1.57	3.04	3.83	4.35
PC-r	2.23	3.53	4.44	4.89	4.73	0.22	0.35	0.71	0.78	0.76	1	1.58	3.19	3.51	3.39
PC-r	1.54	2.29	2.09	2.58	3.24	0.15	0.23	0.33	0.41	0.52	1	1.49	2.17	2.68	3.37
PC-r	1.43	2.13	2.04	2.85	3.45	0.14	0.21	0.33	0.46	0.55	1	1.49	2.28	3.19	3.86
PC-r	1.2	1.87	1.83	2.49	3.02	0.12	0.19	0.29	0.4	0.48	1	1.56	2.44	3.32	4.03
PC-r	1.73	2.9	2.89	4.53	5.46	0.17	0.29	0.46	0.72	0.87	1	1.68	2.67	4.19	5.05
