# Supplementary material for: Culture-space control is effective in promoting haploid cell formation and spermiogenesis in vitro in neonatal mice
Source: Sci Rep. 2023 Jul 31;13:12354. doi: 10.1038/s41598-023-39323-y (PMC10390558; doi:10.1038/s41598-023-39323-y)
Supplement: Supplementary file 13 — Supplementary Figures. [file 41598_2023_39323_MOESM13_ESM.pdf]

Supplementary Figure 1

A

GFP grading scale (PC version)

G1	G2	G3	G4	G5
(1 - 20%)	(21 – 40%)	(41 – 60%)	(61 – 80%)	(81 – 100%)

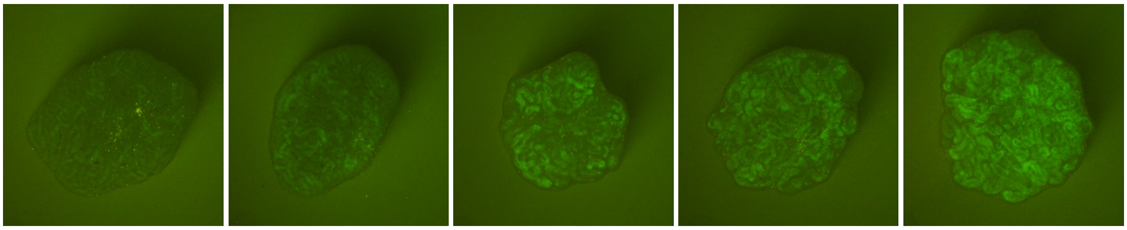

GFP grading scale: GFP expression was classified into six grades, including no GFP expression (grade 0), according to the percentage of GFP expression area observed under a stereomicroscope.

B

Germ cell differentiation grade (GD grade)

	Grade 0	Grade 1	Grade 2	Grade 3	Grade 4
GFP	-	+	+	+	+
PNA (dot shape)	-	-	+	+/-	+/-
PNA (cap shape)	-	-	-	+	+/-
mCherry	-	-	-	-	+
	Representative image	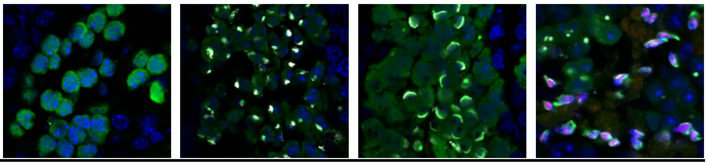			

Germ cell differentiation grade: Seminiferous tubules were classified into five grades based on germ cells they contained. The germ cells were identified by the morphologies and immunostaining patterns of nuclei and acrosomes.
grade 0: Seminiferous tubules that have no germ cells.
grade 1: Seminiferous tubules with germ cells expressing only GFP.
grade 2: Seminiferous tubules with early round spermatids expressing dot-shaped PNA.
grade 3: Seminiferous tubules with late round spermatids expressing cap-shaped PNA.
grade 4: Seminiferous tubules with elongated spermatids (step 11 onward) co-expressing PNA and mCherry.

Supplementary Figure 2

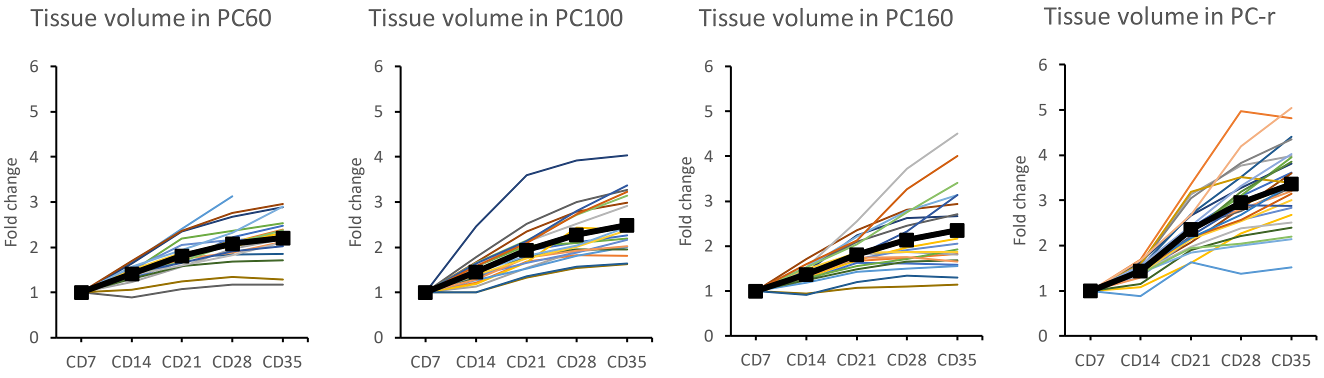

Tissue Volume change: volume change ratio of each sample were plotted along with average (bold black line). Sample numbers were 19, 20, 20, and 26, inPC60, PC100, PC160, and PC-r, respectively.

Supplementary Figure 3

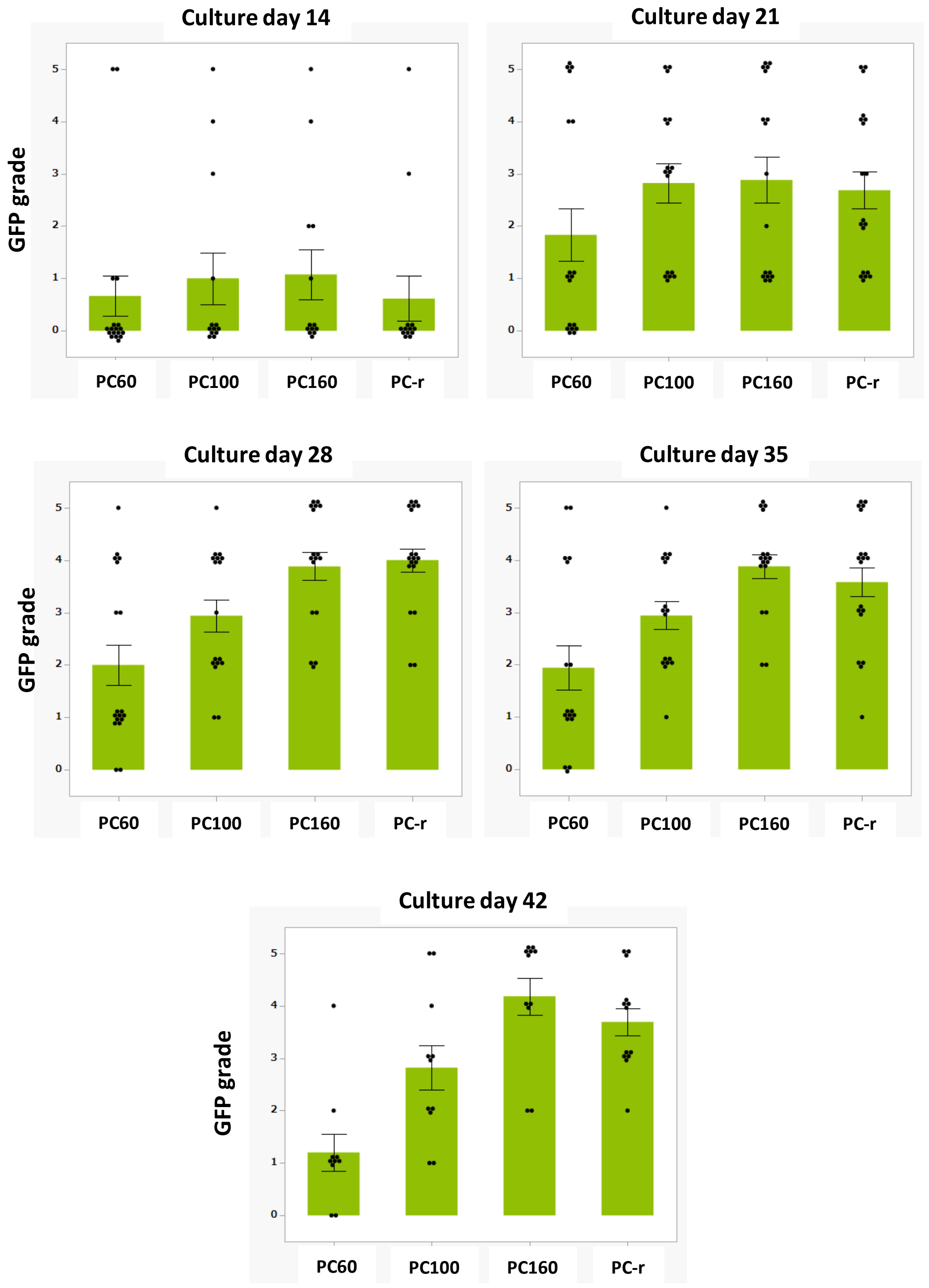

GFP grade comparison among four groups: Data presented in Fig. 2E and 3I were combined, and each data point is shown as a dot.
